# Respiratory viral infections: when and where? A scoping review of spatiotemporal methods

**DOI:** 10.7189/jogh.15.04213

**Published:** 2025-08-04

**Authors:** Jingyi Liang, Daniel Horvath, Saturnino Luz, You Li, Harish Nair

**Affiliations:** 1 Centre for Global Health, Usher Institute, Edinburgh Medical School, University of Edinburgh, Edinburgh, UK; 2 School of Biomedical Sciences, Edinburgh Medical School, University of Edinburgh, Edinburgh, UK; 3 Centre for Medical Informatics, Usher Institute, Edinburgh Medical School, University of Edinburgh, Edinburgh, UK; 4 National Vaccine Innovation Platform, School of Public Health, Nanjing Medical University, Nanjing, China; 5 MRC/Wits Rural Public Health and Health Transitions Research Unit (Agincourt), School of Public Health, University of the Witwatersrand, South Africa

## Abstract

**Background:**

Respiratory viral infections pose a substantial disease burden worldwide. Spatiotemporal techniques help identify transmission patterns of these infections, thereby supporting timely control and prevention efforts. We aimed to synthesise the current state of evidence on quantitative methodologies for investigating the spatiotemporal characteristics of respiratory viral infections.

**Methods:**

We conducted a scoping review using the PRISMA-ScR guidelines. We searched three biomedical bibliographic databases, EMBASE, MEDLINE, and Web of Science, identifying studies that analysed spatiotemporal transmission of viral respiratory infectious diseases (published before 1 March 2023).

**Results:**

We identified 8466 articles from database searches, of which 152 met our inclusion criteria and were qualitatively synthesised. Most included articles (n = 140) were published during the COVID-19 pandemic, with 131 articles specifically analysing COVID-19. Exploratory research (n = 77) investigated the spatiotemporal transmission characteristics of respiratory infectious diseases, focussing on transmission patterns (n = 16), and influencing factors (n = 61). Forecasting research (n = 75) aimed to predict the disease trends using either univariate (n = 57) or multivariate models (n = 18), predominantly using machine learning methods (n = 41). The application of advanced deep learning models (n = 20) in disease forecasting analysis was often constrained by the quality of the available disease data.

**Conclusions:**

There is a growing body of research on spatiotemporal analyses of respiratory viral infections, particularly during the COVID-19 pandemic. The acquisition of high-quality data remains important for effectively leveraging sophisticated models in disease forecasting research. Concurrently, although advanced modelling techniques are widely applied, future studies should consider capturing the complex spatiotemporal interactions in disease trajectory modelling.

Respiratory infectious diseases [1,2], caused by viruses, bacteria, or other pathogens, primarily affect the respiratory system, including the lungs, throat, and airways. Among these, infections of viral aetiology include those related to coronaviruses (*e.g.* COVID-19, MERS-CoV, human influenza viruses, human respiratory syncytial virus (RSV), human metapneumovirus, and human parainfluenza virus), resulting in ailments such as bronchiolitis, influenza-like illnesses, colds, and pneumonia. Additionally, bacterial infections include Streptococcus pneumoniae, Corynebacterium diphtheriae, Bordetella pertussis, and Mycobacterium tuberculosis, among others. Transmission occurs primarily through four modes, *e.g.* direct contact with infected individuals, indirect contact with contaminated surfaces or objects, large respiratory droplets, and fine respiratory aerosols [3].

The transmission of respiratory infectious diseases, including seasonal trends and geographical patterns, is influenced by factors such as population susceptibility and potential exogenous drivers (independent variables that impact disease transmission) such as meteorological conditions [4]. Spatiotemporal analysis plays a vital role in identifying patterns and trends in the transmission of respiratory infectious diseases. Advances in high-performance computing and access to large-scale databases have fueled advancements in spatiotemporal analysis [5]. The rapid development of machine learning (ML) and deep learning (DL) algorithms enables the extraction of substantial insights from complex spatiotemporal data. Spatiotemporal analyses, particularly DL techniques for spatiotemporal data mining, now support applications across various sectors, including environmental monitoring, traffic management, disease surveillance, etc [5-8].

Spatiotemporal analyses have been extensively applied to elucidate and forecast COVID-19 transmission, greatly assisting government responses and preventative endeavours. Ozaki et al. [9] clarified the relationship between population mobility and COVID-19 spread through smartphone global positioning system data, while Nikparvar et al. [10] implemented long short-term memory (LSTM) networks to predict COVID-19 infection and death rates. Indeed, spatiotemporal analyses in respiratory infectious disease research hold promising prospects. The long-term, continuous, and systematic collection of internet data, characterised by its timeliness, abundance, and cost-effectiveness, has proven effective in respiratory infectious disease surveillance [11,12]. Concurrently, the rapid evolution of ML, particularly DL techniques, shows potential for identifying and predicting disease transmission patterns [13].

Previous reviews synthesised spatiotemporal studies to understand the differentiated characteristics of infectious diseases [14]. In contrast, this study synthesises the spatiotemporal methods applied in existing studies. We catalogued the specific methods applied and aligned them with research questions. Furthermore, the COVID-19 pandemic has reshaped the research landscape, leading to the emergence and application of novel approaches, such as ML and DL techniques used in disease forecasting. As such, an up-to-date review to synthesise the current evidence is warranted.

This scoping review aims to analyse in-depth the research data and methods employed in published literature, evaluate the strengths and limitations of existing methods, and identify key research gaps. This review will help improve the understanding of the methods commonly used for spatiotemporal analyses of respiratory viral infections and shed light on future research directions.

## METHODS

We conducted this review following the five-step scoping review protocol [15] and the PRISMA-ScR guidelines [16].

### Search strategy

We conducted literature searches in three bibliographic databases, EMBASE, MEDLINE, and Web of Science, to identify relevant articles published from inception to 1 March 2023. With the assistance of a librarian, we developed the search strategy comprising core concepts related to ‛coronavirus disease 2019/respiratory syncytial virus/influenza virus’, ‛diphtheria/tuberculosis/pertussis’, ‛geographic information systems', ‛spatial analysis/time-series analysis/spatial temporal analysis’ (Tables S2–4 in the **Online Supplementary Document**).

### Inclusion and exclusion criteria

Given the extensive body of literature and the need to ensure accuracy and specificity in this review, we limited our inclusion criteria to studies reporting exclusively on viral respiratory infections within the same taxonomic group, namely influenza virus, SARS-CoV-2, and RSV.

We included articles in English or Chinese that were published from inception until 1 March 2023. The initial eligibility criteria were refined through an iterative process in regular meetings among JL, DH, and HN.

We excluded case-control studies as they focus on investigating risk factors at the individual level. We also excluded studies with data lacking laboratory validation or confirmation, studies with an ambiguous study period, studies that merely conducted descriptive data analysis, and studies concentrated on disease diagnosis or disease evolution.

### Screening procedure

Two reviewers (JL and DH) independently screened the titles and abstracts of the retrieved studies using Covidence (Veritas Health Innovation, Melbourne, Australia), followed by the full texts of the studies selected as potentially eligible by at least one reviewer. The interrater agreement between the two reviewers was 0.64 (n = 4668) for abstract screening and 0.67 (n = 638) for full-text screening. The disagreements between the two reviewers were first discussed and resolved with a third reviewer (HN) if consensus was not reached.

### Data extraction

We extracted and collated the study information using a pre-designed extraction template on Microsoft Excel following an initial pilot. We extracted the following information from included studies: title, first author’s name, year of publication, country of the study, disease, aims, study type, data source, data composition, data granularity, models and algorithms, analysis and programming software, and key findings.

We categorised the studies based on their primary aims into two study types (Table S5 in the **Online Supplementary Document**). Studies aiming to predict future trends in disease transmission were categorised as forecasting analyses (Table S6 in the **Online Supplementary Document**). Studies investigating disease transmission patterns and the relationships between disease transmission and exogenous variables were classified as exploratory analyses (Tables S7 and S8 in the **Online Supplementary Document**).

### Quality assessment

The Critical Appraisal Skills Programme (CASP) checklist was used to assess the quality of the included studies, conducted by an independent reviewer (JL) (Table S9 in the **Online Supplementary Document**).

## RESULTS

**Figure 1:**
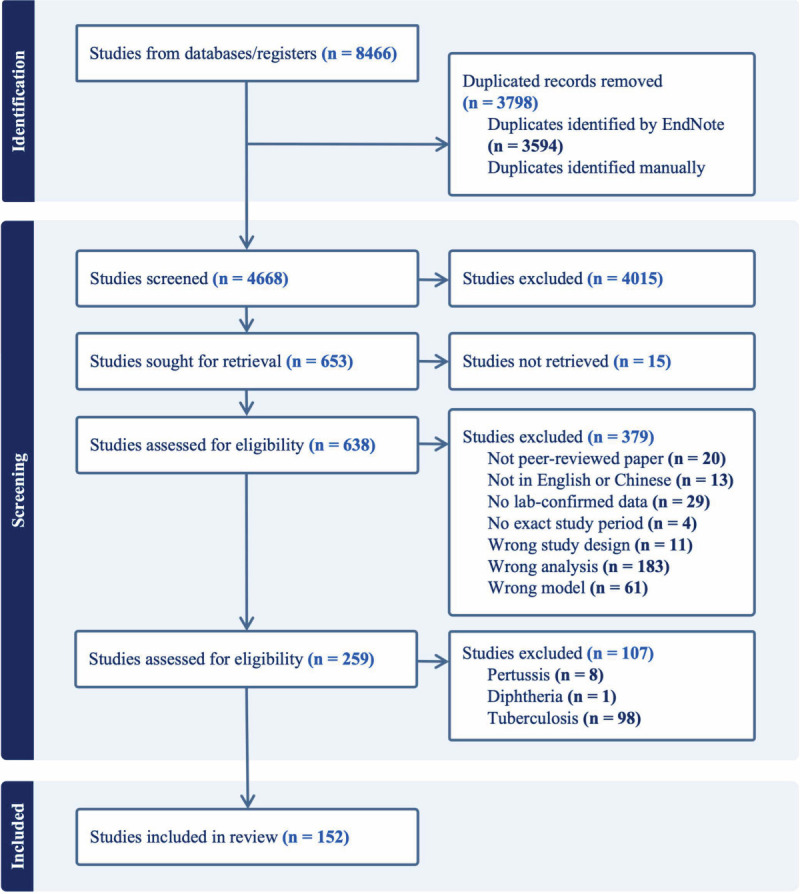
PRISMA flowchart for record retrieval and selection.

From the database search, we retrieved 8466 studies: 2823 from EMBASE, 1917 from MEDLINE, and 3726 from the Web of Science. After removing duplicates and screening titles and abstracts, 638 articles remained for full-text review, with 152 included for analyses (**Figure 1**).

### General characteristics

Of these 152 studies, 86.2% (n = 131) examined COVID-19, while others focussed on RSV (n = 5; 3.3%), influenza (n = 15; 9.9%), or both RSV and influenza (n = 1; 0.6%). Most studies were conducted in a single country (n = 110; 72.4%), 26 (17.1%) covered multiple countries, and 16 (10.5%) had a global scope (Figure S1 in the **Online Supplementary Document**).

We classified the 152 identified studies into exploratory research (n = 77) and forecasting research (n = 75). The exploratory research comprised spatiotemporal pattern analysis (n = 16) and association analysis (n = 61). We observed a shift in research focus with the emergence of the COVID-19 pandemic. Before 2020, exploratory (n = 7) and forecasting (n = 5) research were relatively balanced. In 2020, forecasting research (n = 17) more than doubled in number compared to exploratory research (n = 7). A shift back to the pre-pandemic status quo since 2021, with 53 forecasting studies and 63 exploratory studies recorded.

### General characteristics by disease

Both COVID-19 and influenza studies included forecasting and exploratory analyses. COVID-19 studies spanned a range of scales, from national to global, with data collection periods varying from less than one year to more than two years. Influenza studies were primarily single-country analyses, with data ranging from under one year to over a decade. RSV research was limited to exploratory, single-country analyses, covering data from under one year to over a decade (Figure S2 in the **Online Supplementary Document**).

### Data characteristics

We summarised the data characteristics in the included studies (Figure S3 in the **Online Supplementary Document**). Time-series data, which involves one-dimensional measurements taken over time, appeared in 27% (n = 41) of the studies. Panel data, which involves multi-dimensional data comprising measurements over time at multiple locations, were used in 73% (n = 111), with over half (n = 56) incorporating spatial coordinates. Regarding spatial granularity, nationwide data were analysed in 64 (42.1%) and finer-scale data in 88 (57.9%) studies. For temporal granularity, most studies (n = 128; 84.2%) analysed daily data, with fewer in weekly (n = 15; 9.9%), monthly (n = 8; 5.3%), or yearly (n = 1; 0.6%). The duration of the studies varied, with 113 (74.3%) lasting less than a year, 24 (15.8%) lasting 1–2 years, and 3 (2%) studies extending over 10 years.

### Methods and algorithms

**Figure 2:**
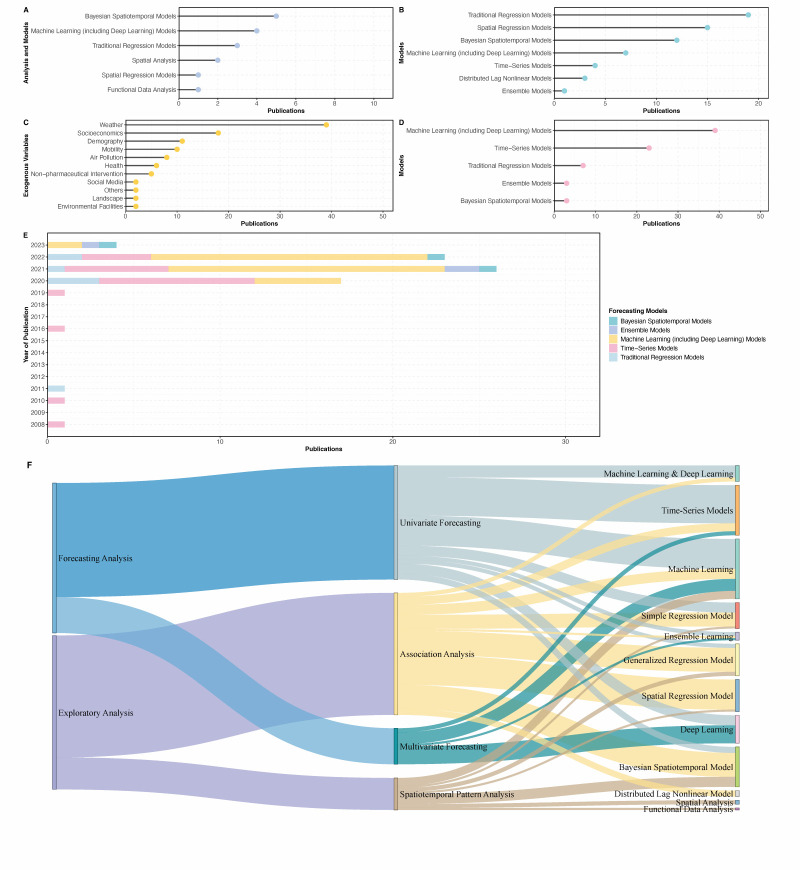
Characteristics of analyses and algorithms in the identified scenario literature. **Panel A.** Analysis and models applied in spatiotemporal pattern analyses. **Panel B.** Models applied in association analyses. **Panel C.** Exogenous variables investigated in association analyses. **Panel D.** Models applied in forecasting analyses. **Panel E.** Models applied in forecasting analyses by year. **Panel F.** Sankey diagram visualisation of study categorisation. The weight of each edge is proportional to the number of studies. Edges are colored based on analysis types.

We provided a data summary of research objectives, methods, and algorithms in **Figure 2**.

### Forecasting analysis

**Figure 3:**
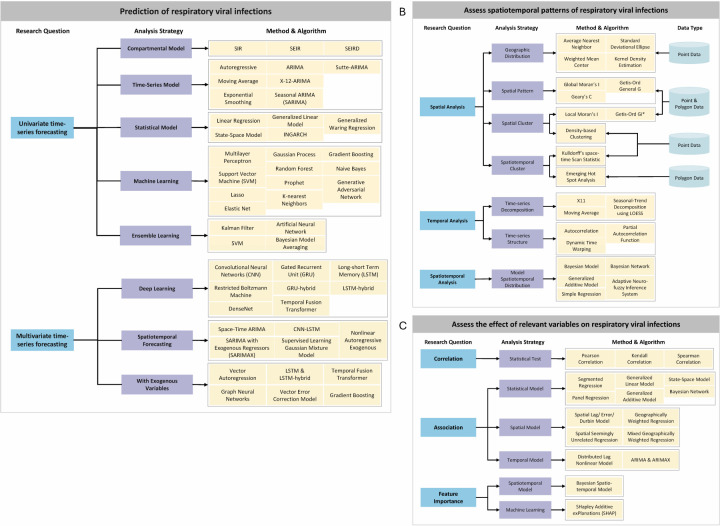
Diagram of research question, methods, and algorithms in current research. **Panel A.** Diagram of research question, methods, and algorithms in forecasting analyses. **Panel B.** Diagram of research question, methods, and algorithms in spatiotemporal pattern analyses. **Panel C.** Diagram of research question, methods, and algorithms in association analyses.

Forecasting analysis (**Figure 3**) identifies temporal disease patterns and predicts future trends, including: absolute counts (cases or deaths), indicators (incidence, prevalence, mortality), and key time points (onset or peak). We categorised forecasting methods as univariate or multivariate and discussed standard models by ascending complexity.

Univariate forecasting relies solely on historical disease information to predict future trajectories. Time-series models are dynamic systems that are identified to fit a given signal or time-series data [17]. The Autoregressive Integrated Moving Average (ARIMA), Exponential Smoothing, and Holt-Winters models excel at capturing temporal patterns such as long-term trends, with relatively low complexity. Sulasikin et al. [18] applied ARIMA to forecast COVID-19 confirmed cases. Sophisticated variants, such as X-12-ARIMA [19], can effectively adjust for seasonal fluctuations [20]. ML is broadly defined as computational methods using experience to make accurate predictions [21]. Known for managing complex nonlinear relationships, ML has demonstrated effectiveness in predicting disease trends. The Prophet model [22-25] has been widely used to predict potential COVID-19 outbreaks. Other ML models aiding in disease trend predictions include the multilayer perceptron [26-29], support vector machine [25,26,30-33], tree-based models including random forest [28,30,33-37], gradient boosting [34], XGBoost [32,35], and regularised regression models [38], including Lasso and Elastic Net. Deep learning [39], a subset of ML, excels at processing large data sets to identify intricate patterns, yielding accurate forecasts. Among them, recurrent neural networks [40] are designed to handle sequential data. Similar variants, such as LSTM [33,41-45], and gated recurrent units [38,40], have been extensively applied in estimating COVID-19 outbreaks. Ensemble learning [46], for example, Bayesian model averaging [41], can further enhance prediction stability and accuracy by aggregating results from multiple models.

Multivariate forecasting analysis combines auxiliary variables with historical disease data to forecast disease trends. Specifically, spatiotemporal forecasting leverages disease and spatial/movement data from multiple locations to predict regional trends concurrently. Traditional regression models, such as mixed-effects generalised linear model [47], have been applied to predict the spatiotemporal progression of COVID-19. Time-series models, such as multivariate space-time ARIMA [48], are specifically tailored for analysing the spatiotemporal spread of diseases. Moreover, DL models further enhance forecasting performance. For instance, the convolutional neural network-LSTM architecture [49], where the convolutional neural networks learn spatial dependencies and the LSTM block captures temporal dependencies, provides insights into the spatiotemporal spreading of COVID-19.

### Spatiotemporal pattern analysis

Spatiotemporal pattern analysis explores how disease was transmitted through space and time to reveal transmission dynamics (**Figure 3**). It involves:

− Visualisation and interpretation of disease spreading across temporal and geographical dimensions.
− Quantification of spatial patterns and variations of disease transmission, assessing the relationship between disease transmission and geographical locations.
− Identification of regions that exhibit elevated vulnerability to infections.
− Simulation of disease trajectories.

Spatial distribution analysis measures the geographical dispersion of diseases. It identifies the epicentre and quantifies the central tendency, dispersion, and directional trends. Methods such as weighted mean centre analysis and directional distribution analysis are instrumental in pinpointing epicentres and monitoring propagation [50].

Spatial pattern analysis evaluates the spatial relationship between incidence, mortality, and other metrics across regions. Global autocorrelation analysis assesses the spatial randomness of disease distribution, evaluating the degree of clustering or dispersion within an area. Commonly employed measures include Global Moran’s I [50-52] and Getis-Ord General G [53]. Local measures of spatial autocorrelation describe spatial variations in different regions of the study area, assuming spatial heterogeneity. Anselin Local Moran's I [54] and Getis-Ord Gi* [50,51,53] are two commonly used methods to locate high- and low-risk disease clusters in a particular region.

Spatiotemporal pattern mining techniques identify trends and clusters in disease spread across time and regions. Techniques like emerging hotspot analysis [52] and Kulldorff’s space-time scan statistic [54] have been applied to identify regions with high risk (hotspots) and low risk (coldspots) over time.

Modelling techniques simulate the disease trajectory. Regression models [51,55,56], from simple linear to generalised additive models, can effectively predict the disease trajectory over time. Bayesian spatiotemporal models [54,57-60] incorporate spatial information, offering a more dynamic portrayal of disease transmission. Furthermore, hybrid models, such as a two-stage modelling approach proposed by Zheng et al. [61], further assess the fine-scale spatial variations in epidemic timing.

### Association analysis

Association analysis (**Figure 3**) quantifies exogenous drivers of disease transmission across specific periods and areas, including:

− Identify and evaluate how influential factors are related to disease transmission.
− Assess how these relationships vary over time and space.

Common variables include demographic (n = 20) and socioeconomic (n = 24) conditions, health status (n = 6), meteorological conditions (n = 43), air pollution (n = 8), mobility patterns (n = 12), and lockdown policies (n = 5).

Several methods have been applied to identify influential factors. Correlation tests preliminarily screen variables that correlate with disease transmission. ML and DL techniques (n = 5) offer new perspectives. Variables are considered influential when including them would enhance the model performance. Meanwhile, tree-based models and SHapley Additive exPlanations [62-64] can assess the feature importance to identify determinants.

Traditional regression analysis, employing regression models presuming spatial and temporal independence [65,66], quantifies how changes in external factors affect disease transmission. Sophisticated modelling techniques are essential to address the spatial and temporal dependencies inherent in disease data. Spatial regression models, including spatial lag model [67-70], spatial error model [68-70], and spatial Durbin model [68,71], are designed to tackle spatial dependencies, enhancing the precision of the analysis. To assess local variations in relationships, geographically weighted regression is advantageous. Huang et al. [72], applied geographically weighted regression to investigate the relationships between environmental factors and COVID-19 cases. Time-series models, accounting for temporal dependencies, delineate how external variables influence disease transmission over time. Ademu et al. [73] and Chien et al. [74] used generalised additive models and distributed nonlinear lag models, respectively, to examine the time-delayed effects of environmental changes on COVID-19. Bayesian spatiotemporal models [75-86] integrate temporal trends, spatial dependencies, and spatiotemporal variability, offering a comprehensive view of how associations evolve.

## DISCUSSION

Our scoping review is the first, to our knowledge, to systematically investigate studies focussed on spatiotemporal transmission of viral respiratory infectious diseases, including COVID-19, influenza, and RSV. We categorised 152 articles by research focus, summarising the problems addressed, methodologies employed, and specific algorithms applied. Our findings suggest that spatiotemporal analyses can provide a comprehensive understanding of the epidemic of respiratory viral infections from both temporal and spatial perspectives. This review emphasises the significance of spatiotemporal analyses in understanding and predicting disease transmission.

Of the 152 studies, the earliest one was published in 2008 [87], and the latest ones were published in 2023. Research on the spatiotemporal analyses of respiratory viral infections has grown markedly since 2020, after the COVID-19 outbreak. Forecasting analyses surged during the early phase of the pandemic, driven by an urgent need to control and prevent the COVID-19 spread. Accurate forecasts can cost-effectively support the allocation of medical resources and inform the formulation of effective prevention strategies to manage such a global health crisis. Additionally, available data from the early pandemic, with limited features and durations (under one year), were suited for short-term forecasting. In contrast, identifying long-term trends and relationships from such short-term data was inadequate, possibly yielding inaccurate conclusions.

As for forecasting analyses of respiratory viral infections, we observed no differences in the methods applied to analyse different geographical regions worldwide. For example, we observed no significant differences in modelling choices used to analyse COVID-19 trends in Asia and Africa. We found that methods have evolved markedly over time, particularly after the COVID-19 pandemic in 2020. Prior to 2020, most studies employed time-series and simple regression models. Afterwards, advanced techniques have gained prominence, including ML, DL, and Bayesian spatiotemporal models. Real-time, global COVID-19 surveillance systems now provide high-continuity, fine-grained (daily or hourly) data, enabling complex models to simulate nonlinear relationships more effectively. Simpler methods (*e.g.* ARIMA and linear regression) often underfit large-scale epidemic data and fail to capture intricate spatiotemporal patterns. Although DL architectures, such as transformers [88], excel at processing large-scale data, they can overfit or become unstable when the data are sparse or imbalanced [29]. Rigorous evaluation of model generalisability via cross-validation, holdout testing, and bootstrapping is therefore essential to select optimal models according to the data set's characteristics.

The diversity of forecasting methods and the flexible adaptation of data enhance the accuracy of predictions for future trends, informing disease control and prevention. Nonetheless, the identified studies exhibit several limitations. First, the absence of comparative benchmarks. Some studies, like Khan et al. [89], did not conduct comparative or benchmark experiments to evaluate the model’s performance. Second, the use of short-term data for long-term forecasts. Such cases, as in Arun et al. [43], may result in biased and unstable forecast outcomes. Third, inadequate data segmentation. Qureshi et al. [29], applied year-round data, without segmenting data based on its characteristics, to develop tailored models. Additionally, neglect spatial dependencies in multi-regional forecasting. Some multi-country or multi-regional studies [10,32], only using time-series models, overlooking spatial effects, may diminish the predictive accuracy. Lastly, the underutilisation of advanced methods. Constrained by data availability and overfitting concerns, some studies [90-92] relied solely on time-series models, such as ARIMA, without exploring more sophisticated models.

Exploratory analyses of disease spatiotemporal patterns encompass three aspects: temporal, spatial, and spatiotemporal, with the latter two most emphasised. Temporal pattern analysis often integrates with disease prediction efforts, uncovering periodic or cyclical disease features to enhance prediction accuracy. Spatial pattern analysis leverages geographic information systems tools to investigate the geographical disease distributions. Among them, ArcGIS (n = 18) emerged as the most frequently used, followed by GeoDa (n = 4) and QGIS (n = 3). Spatiotemporal pattern analysis elucidates the dynamics of disease transmission. Advanced tools, such as SaTScan [54,93], and sophisticated spatiotemporal models help identify and manage disease outbreaks more precisely. However, reviewed studies exhibit two main limitations. First, descriptive visualisation only. Some studies, such as those by Al-Kindi et al. [50], merely visualised the disease spread patterns. Second, neglect the time-space interactions. Some studies, such as Bag et al. [51], simulated trajectories without jointly modelling temporal and spatial dependencies, which hinders a comprehensive understanding of disease dynamics.

Various regression models, including traditional, spatial, and spatiotemporal, are widely applied to identify factors influencing disease transmission. However, these methods come with several limitations. First, ecological fallacy. Extrapolating findings from the regional level to individuals can be misleading [83,86,94-96]. Second, while associations between influencing factors and disease transmission can be established, determining causality remains challenging [78,93,97]. Third, overlooking economic, demographic, and social factors, crucial to disease transmission risk, introduces potential biases [73,98]. Additionally, there is a lack of subgroup analyses. Inadequate understanding of disease risk variations across different groups (*e.g.* sociodemographic groups) [73]. Lastly, the limited generalisability. Reliance on short-term data from narrow geographic areas restricts the broader applicability of findings [80,86].

Data plays a crucial role in research. In the identified 152 publications, common data-related issues exist. First, data quality issues, including inconsistent disease definitions, heterogeneous data collection methods, incomplete data, particularly during the early stages of the COVID-19 pandemic, and limited availability of data on potential confounding factors. Second, challenges in multi-source data fusion. Integrating data from different sources, particularly when these sources operate on varying time scales, presents significant complexities. Third, data smoothing trade-offs. Data smoothing mitigates reporting lags and reflects incubation periods while reducing the data precision. Additionally, there is limited data volume. Small data sets restrict the research depth and generalisability, especially evident in early studies of COVID-19. Considering that, synthetic data offers a possible solution. Synthetic data, simulated to mirror statistical patterns from real-world data, fills gaps in small or unbalanced data sets and avoids privacy risk. An Austrian study [99], demonstrated that synthetic data of COVID-19 cases successfully provided additional information on untested cases to support more comprehensive epidemiological analysis.

This review provides a detailed synthesis of quantitative methodologies for investigating the spatiotemporal aspects of viral respiratory infections, establishing the groundwork for future research. Our study acknowledges several limitations. First, we limited our search to studies published until 1 March 2023. Second, we only considered articles published in English and Chinese. We excluded 13 articles based on language, although they did fulfil other eligibility criteria. Third, the focus of this review was limited to three specific respiratory viral infections, thereby excluding other respiratory infections, including central bacterial infections such as tuberculosis, diphtheria, and whooping cough.

We outline several recommendations for future research. First, future work should broaden the scope of influenza and RSV research. For influenza, employing multivariate time-series forecasting with external variables is advised to enhance prediction accuracy. RSV research should integrate both forecasting and spatiotemporal pattern analysis to deepen insights into transmission dynamics. Second, future work should also prioritise improving data quality and richness. This includes strengthening surveillance systems to produce high-quality, large-scale data sets, acquiring high-quality multimodal health data from sources such as wearable devices and mobile health applications, and exploring the generation and application of synthetic data. Third, future work optimises the effectiveness of spatiotemporal analyses. Advanced methods that incorporate spatial and temporal information enhance disease diagnosis, monitoring, and prevention.

## CONCLUSIONS

Advanced machine learning and deep learning techniques have enriched the spatiotemporal analyses of respiratory viral infections. Currently, small data sets constrain the performance and generalisability of deep learning models. Forthcoming studies should prioritise the acquisition of extensive, high-quality data sets via global surveillance networks or synthetic data generation. Subsequently, future work should effectively leverage sophisticated models while balancing interpretability and predictive accuracy. Specifically, future methodological developments should capture intricate spatiotemporal interactions in disease transmission and control, accounting for potential confounders, to elucidate the determinants of transmission.

## Additional Material


Online Supplementary Document
