# Supplementary material for: Respiratory viral infections: when and where? A scoping review of spatiotemporal methods
Source: J Glob Health. 2025 Aug 4;15:04213. doi: 10.7189/jogh.15.04213 (PMC12319400; doi:10.7189/jogh.15.04213)
Supplement: Online Supplementary Document [file jogh-15-04213-s001.pdf]

Supplement to: Liang J, Horvath D, Luz S, Li Y, Nair H. Respiratory viral infections: when and where? A scoping review of spatiotemporal methods. J Glob Health. 2025;15:04213.

Table S1. List of abbreviation and acronyms used in the paper

Abbreviation	Definition	Abbreviation	Definition
ANFIS	Adaptive Neuro-fuzzy Inference System	LM	Linear Model
ANN	Artificial Neural Network	LR	Logistic Regression
AR	Autoregressive Model	LSTM	Long-Short Term Memory
ARIMA	Autoregressive Integrated Moving Average	MA	Moving Average
ARIMAX	Autoregressive Integrated Moving Average with Explanatory Variable	MAE	Mean Absolute Error
ARMA	Autoregressive Moving Average	MAPE	Mean Absolute Percentage Error
BiLSTM	Bi-directional LSTM	MLP	Multilayer Perceptron
BMA	Bayesian Model Averaging	NARANN	Nonlinear Autoregressive Artificial Neural Network
CNN	Convolutional Neural Networks	NARX	Nonlinear Autoregressive Exogenous Model
DT	Decision Tree	NB	Naïve Bayes
ED	Encoder-Decoder	NB INGARCH	Negative Binomial Integer Autoregressive Conditional Heteroscedasticity
EEMD	Ensemble Empirical Mode Decomposition	PCA	Principal Component Analysis
EnKF	Ensemble Kalman Filter	RBM	Restricted Boltzmann Machine
ES	Exponential Smoothing	RF	Random Forest
GAM	Generalized Additive Model	RMSE	Root Mean Square Error
GAN	Generative Adversarial Network	RNN	Recurrent Neural Network
GBM	Gradient Boosting Machines	SARIMA	Seasonal Autoregressive Integrated Moving Average
GLARMA	Generalized Linear Autoregressive Moving Average	SARIMAX	Seasonal Autoregressive Integrated Moving Average with Explanatory Variable
GLM	Generalized Linear Regression	SEIR	Susceptible-Exposed-Infected-Recovered Model
GNN	Graph Neural Network	SEIRD	Susceptible-Exposed-Infected-Recovered-Dead Model
GP	Gaussian Process	SGLMM	Spatial Generalised Linear Mixed Models
GRU	Gated Recurrent Unit	SHAP	SHapley Additive exPlanations
GWR	Geographically weighted Regression	SIR	Susceptible-Infected-Recovered Model
GWRM	Generalized waring regression Model	STARIMA	Spatial Time-Autoregressive Integrated Moving Average
HC	Hierarchical Clustering	SVM	Support Vector Machine
HW	Holt-Winters Forecasting	VAR	Vector Autoregressive Model
KNN	K-Nearest Neighbors	XGBoost	eXtreme Gradient Boosting

Table S2. Search strategy of Embase

Terms	Number of Result
exp Human respiratory syncytial virus/	8314
exp respiratory syncytial virus infection/di, dm, ep, pc [Diagnosis, Disease Management, Epidemiology, Prevention]	2309
rsv.mp.	19594
1 or 2 or 3	24111
exp Influenza A virus/	19840
exp Influenza B virus/	2365
exp Influenza A H1N1/	6681
exp Influenza A H3N2/	3288
5 or 6 or 7 or 8	20362
exp coronavirus disease 2019/di, dm, ep, pc [Diagnosis, Disease Management, Epidemiology, Prevention]	62200
exp diphtheria/di, dm, ep, pc [Diagnosis, Disease Management, Epidemiology, Prevention]	4902
exp pertussis/di, dm, ep, pc [Diagnosis, Disease Management, Epidemiology, Prevention]	7720
exp tuberculosis/di, dm, ep, pc [Diagnosis, Disease Management, Epidemiology, Prevention]	75001
4 or 9 or 10 or 11 or 12 or 13	188587
(transmission* or case* or incidence* or mortality or morbidity).mp. [mp=title, abstract, heading word, drug trade name, original title, device manufacturer, drug manufacturer, device trade name, keyword heading word, floating subheading word, candidate term word]	9541936
14 and 15	102538
exp geographic information system/ or Geographic Information System*.mp. or exp geographic distribution/	165890
((Statistic* or machine or deep) adj3 (learn* or model*)).mp. [mp=title, abstract, heading word, drug trade name, original title, device manufacturer, drug manufacturer, device trade name, keyword heading word, floating subheading word, candidate term word]	371996
((spati* or space) adj3 (analy* or model* or regress* or cluster* or pattern*)).mp. [mp=title, abstract, heading word, drug trade name, original title, device manufacturer, drug manufacturer, device trade name, keyword heading word, floating subheading word, candidate term word]	99813
(time-series adj3 analy*).mp. [mp=title, abstract, heading word, drug trade name, original title, device manufacturer, drug manufacturer, device trade name, keyword heading word, floating subheading word, candidate term word]	44047
17 or 18 or 19 or 20	659824
16 and 21	3743

Table S3. Search strategy of MEDLINE

Terms	Number of Result
exp Respiratory Syncytial Viruses/an, cl, im [Analysis, Classification, Immunology]	3602
exp Respiratory Syncytial Virus Infections/cl, di, ep, mo, pc, tm [Classification, Diagnosis, Epidemiology, Mortality, Prevention & Control, Transmission]	4755
rsv.mp.	13063
1 or 2 or 3	15438
exp Influenza A virus/an, cl, di, ep, gd [Analysis, Classification, Diagnosis, Epidemiology, Growth & Development]	5065
exp Influenza B virus/an, cl, ep, gd [Analysis, Classification, Epidemiology, Growth & Development]	492
exp Influenza, Human/cl, di, dg, ep, mo, pc, sn, tm [Classification, Diagnosis, Diagnostic Imaging, Epidemiology, Mortality, Prevention & Control, Statistics & Numerical Data, Transmission]	39200
exp influenza a virus, h1n1 subtype/ or exp influenza a virus, h3n2 subtype/	19645
5 or 6 or 7 or 8	51426
exp COVID-19/cl, di, dg, ep, mo, pc, tm [Classification, Diagnosis, Diagnostic Imaging, Epidemiology, Mortality, Prevention & Control, Transmission]	77030
exp SARS-CoV-2/cl [Classification]	473
10 or 11	77159
exp Diphtheria/an, cl, di, dg, ep, mo, pc, sn, tm, td [Analysis, Classification, Diagnosis, Diagnostic Imaging, Epidemiology, Mortality, Prevention & Control, Statistics & Numerical Data, Transmission, Trends]	3092
exp Whooping Cough/cl, di, dg, ec, ep, mo, pc, sn, tm [Classification, Diagnosis, Diagnostic Imaging, Economics, Epidemiology, Mortality, Prevention & Control, Statistics & Numerical Data, Transmission]	5883
Pertussis.mp.	30696
exp Tuberculosis, Pleural/ or exp Tuberculosis, Pulmonary/	79792
4 or 9 or 12 or 13 or 14 or 15 or 16	254921
(transmission* or case* or incidence* or mortality or morbidity).mp. [mp=title, book title, abstract, original title, name of substance word, subject heading word, floating sub-heading word, keyword heading word, organism supplementary concept word, protocol supplementary concept word, rare disease supplementary concept word, unique identifier, synonyms, population supplementary concept word, anatomy supplementary concept word]	6728057
17 and 18	90902
exp Geographic Information Systems/	9218
((Statistic* or machine or deep) adj3 (learn* or model*)).mp. [mp=title, book title, abstract, original title, name of substance word, subject heading word, floating sub-heading word, keyword heading word, organism supplementary concept word, protocol supplementary concept word, rare disease supplementary concept word, unique identifier, synonyms, population supplementary concept word, anatomy supplementary concept word]	203209
GIS.mp.	8330
((spati* or space) adj3 (analy* or model* or regress* or cluster* or pattern*)).mp. [mp=title, book title, abstract, original title, name of substance word, subject heading word, floating sub-heading word, keyword heading word, organism supplementary concept word, protocol supplementary concept word, rare disease supplementary concept word, unique identifier, synonyms, population supplementary concept word, anatomy supplementary concept word]	68742
(time-series adj3 (analy* or trend*)).mp. [mp=title, book title, abstract, original title, name of substance word, subject heading word, floating sub-heading word, keyword heading word, organism supplementary concept word, protocol supplementary concept word, rare disease supplementary concept word, unique identifier, synonyms, population supplementary concept word, anatomy supplementary concept word]	11416
20 or 21 or 22 or 23 or 24	287971
19 and 25	2976

Table S4. Search strategy of Web of Science

Terms	Number of Result
TI=("Wuhan coronavirus" OR "COVID19*" OR "COVID-19*" OR "COVID-2019*" OR "coronavirus disease 2019" OR "SARS-CoV-2" OR "2019-nCoV" OR "2019 novel coronavirus" OR "severe acute respiratory syndrome coronavirus 2" OR "2019 novel coronavirus infection" OR "coronavirus disease 2019" OR "coronavirus disease-19" OR "SARS-CoV-2019" OR "SARS-CoV-19") OR AB=("Wuhan coronavirus" OR "COVID19*" OR "COVID-19*" OR "COVID-2019*" OR "coronavirus disease 2019" OR "SARS-CoV-2" OR "2019-nCoV" OR "2019 novel coronavirus" OR "severe acute respiratory syndrome coronavirus 2" OR "2019 novel coronavirus infection" OR "coronavirus disease 2019" OR "coronavirus disease-19" OR "SARS-CoV-2019" OR "SARS-CoV-19") OR KP=("Wuhan coronavirus" OR "COVID19*" OR "COVID-19*" OR "COVID-2019*" OR "coronavirus disease 2019" OR "SARS-CoV-2" OR "2019-nCoV" OR "2019 novel coronavirus" OR "severe acute respiratory syndrome coronavirus 2" OR "2019 novel coronavirus infection" OR "coronavirus disease 2019" OR "coronavirus disease-19" OR "SARS-CoV-2019" OR "SARS-CoV-19")	394783
TI=("respiratory syncytial virus*" OR RSV) OR AB=("respiratory syncytial virus*" OR RSV) OR KP=("respiratory syncytial virus*" OR RSV)	26005
TI=("Human Influenza Virus*" OR "Influenza Virus A" OR "Influenza Virus B" OR "Influenza*") OR AB=("Human Influenza Virus*" OR "Influenza Virus A" OR "Influenza Virus B" OR "Influenza*") OR KP=("Human Influenza Virus*" OR "Influenza Virus A" OR "Influenza Virus B" OR "Influenza*")	160080
TI=(Diphtheria) OR AB=(Diphtheria) OR KP=(Diphtheria)	16737
TI=(Pertussis OR "Whooping Cough") OR AB=(Pertussis OR "Whooping Cough") OR KP=(Pertussis OR "Whooping Cough")	30944
TI=("Tuberculosis Pulmonary"OR "Tuberculosis Pleural" OR "Lung Tuberculosis" OR TB) OR AB=("Tuberculosis Pulmonary"OR "Tuberculosis Pleural" OR "Lung Tuberculosis" OR TB) OR KP=("Tuberculosis Pulmonary"OR "Tuberculosis Pleural" OR "Lung Tuberculosis" OR TB)	97102
TI=(Transmission* OR Incidence* OR Case* OR Mortality OR Morbidity) OR AB=(Transmission* OR Incidence* OR Case* OR Mortality OR Morbidity) OR KP=(Transmission* OR Incidence* OR Case* OR Mortality OR Morbidity)	8962307
#1 OR #2 OR #3 OR #4 OR #5 OR #6	699212
#7 AND #8	200929
TI=("Statistic* learn*" OR "machine learn*" OR "deep learn*" OR "statistic* model*") OR AB=("Statistic* learn*" OR "machine learn*" OR "deep learn*" OR "statistic* model*") OR KP=("Statistic* learn*" OR "machine learn*" OR "deep learn*" OR "statistic* model*")	435796
TI=("Geographic Information System*" OR GIS) OR AB=("Geographic Information System*" OR GIS) OR KP=("Geographic Information System*" OR GIS)	95515
TI=("spati* regress*" OR "spati* analy*" OR "spati* model*" OR "spati* pattern*" OR "spati* distribution*" OR"spati* cluster*") OR AB=("spati* regress*" OR "spati* analy*" OR "spati* model*" OR "spati* pattern*" OR "spati* distribution*" OR"spati* cluster*") OR KP=("spati* regress*" OR "spati* analy*" OR "spati* model*" OR "spati* pattern*" OR "spati* distribution*" OR"spati* cluster*")	247948
TI=("time-series analy*" OR "temporal analy*" OR "time-series model*" OR "temporal model*") OR AB=("time-series analy*" OR "temporal analy*" OR "time-series model*" OR "temporal model*") OR KP=("time-series analy*" OR "temporal analy*" OR "time-series model*" OR "temporal model*")	48603
#10 OR #11 OR #12 OR #13	802425
#9 AND #14	5794

Table S5. Study characteristics of the identified scenario literature (n = 152).

Study Type	Study Aims	No. Publications
Exploratory Research	Assess the effect of exogenous variables on disease transmission	61
	Assess the spatiotemporal patterns of disease transmission	16
Forecasting Research	Prediction of disease transmission	57
	Prediction of disease transmission with exogenous variables	18

Table S6. A list of published studies in forecasting research (n = 75)

Ref	Aims & Objectives	Methods	Algorithms & Models	Model Performance	Key Findings
[1]	Model and predict the spread and outbreak of COVID-19 with uncertainties using Bayesian machine learning methods.	Deep Learning	Bayesian-LSTM	The LSTM-INLA (RMSE = 5.51) model had the lowest RMSE prediction compared to LSTM (RMSE = 6.07) and INLA (RMSE = 14.24) models.	A Bayesian model informed by a neural network method could be generally able to predict the number of cases of COVID-19 in both space and time, with the human mobility factor having a strong influence on the model, together with the number of infections and deaths in nearby areas.
[2]	Predict the number of new cases and new deaths due to COVID-19 and forecast them within up to 6 days.	Machine Learning	MLP-ANN	The MLP network achieved good results with just one hidden layer for forecasting cases (MAE = 3718).	The MLP-ANN model can effectively describe and predict for up to six days the behavior of time series related to the number of infected cases and deaths by COVID-19.
[3]	Identify the meteorological risk factors for COVID-19 incidence and compare the predictive accuracy of ARIMAX and XGBoost for COVID-19 incidence in SAARC countries.	Machine Learning	ARIMAX, XGBoost	XGBoost model outperformed ARIMAX models in predictions of COVID-19 cases in SAARC countries, with achieving lower MAE, MAPE, and RMSE.	The maximum temperature had a positive impact on COVID-19 transmission in Afghanistan and India. Surface pressure had a positive influence on Pakistan and Sri Lanka. The XGBoost model can help improve the prediction of COVID-19 cases in SAARC countries over the ARIMAX model.
[4]	Predict and prevent COVID-19 epidemics in the USA.	Ensemble Learning	RF, LightGBM, XGBoost, SHAP	The accuracy of prediction was as follows in descending order: LightGBM (MAE = 8814.68, MAPE = 6.27%), XGBoost (MAE = 9804.77, MAPE = 6.27%), and RF (MAE = 5140.97, MAPE = 9.52%).	Compared with the base learners, the ensembles provided better accuracy in forecasting the COVID-19 trend in the USA. Vaccination, wearing a mask, less mobility, and appropriate government intervention measures could effectively slow the incidence rate.
[5]	Propose an encoder-decoder BiLSTM deep learning model to forecast the COVID-19 confirmed cases, recovered cases, and death cases.	Machine & Deep Learning	AIRMA, SVM, Elastic Net, Lasso, LSTM, BiLSTM, GRU, ED-BiLSTM	Encoder-Decoder BiLSTM forecasting model outperformed the other models, achieving the lowest RMSE, MAE and MSE and the highest R^2(0.99).	The proposed model performed best. It may help policymakers improve pandemic spread control and be generalized for other time series forecasting tasks.
[6]	Analyze and forecast COVID-19 daily confirmed new cases in Saudi Arabia.	Time-Series	ARIMA, log-liner Poisson AR	The log-liner Poisson Autoregressive model outperformed the ARIMA model in this study (MAE = 5210.21, RMSE = 72.10, MAPE = 38.02).	A log-linear autoregressive Poisson model is more accurate than the ARIMA model, considering that pandemic evolution data is count data.
[7]	Propose a simple yet efficient machine learning model that addresses the problem of nowcasting in a way that is easily understood by non-experts.	Machine Learning	RF, Bayesian Model	The predictions by the RF were relatively closer to the true values than those generated using the Bayesian model estimates.	The predictions from this simple data-driven method compare favorably both in quality and computational burden to those obtained from the state-of-the-art hierarchical Bayesian model employing a complex statistical algorithm.
[8]	Predict COVID-19 incidence at the county level in the contiguous US over 1-, 2-, 3-, and 4-week forecast horizons.	Deep Learning	LSTM, LSTM-Hybrid	COVIDhub-ensemble performed best (MAE = 78.77) at week 1, while for 2, 3, and 4 weeks prediction, COVID-LSTM (2 weeks: MAE = 110.97, 3 weeks: MAE = 121.46, 4 weeks: MAE = 133.22) performed best.	The data-driven, spatiotemporal forecasting using deep learning was more accurate than the COVID-19 Hub Ensemble of multiple models at predicting county-level incidence over 2-, 3-, and 4-week forecast horizons.
[9]	Forecast COVID-19 confirmed cases at the county level for the United States.	Ensemble Learning	AR,LSTM, EnKF, BMA	The Bayesian ensemble model surpassed other models in the CDC's COVID-19 Forecast Hub, especially at extended forecast periods.	The Bayesian ensemble proposed in this study is at least as good as the individual methods, while each individual method contributes significantly to different spatial regions and time points.
[10]	Predict COVID-19 epidemics using machine learning regressors.	Machine Learning	LM, Polynomial Regression, RF, DT	The polynomial regression performed better in forecasting (predicted cases = 181,723,467, actual confirmed cases = 181,722,790).	The polynomial regression technique can predict future cases better.
[11]	Evaluate, optimize, and fine-tune state-of-the art prediction models in order to enhance their performance and automate their functions as much as possible.	Time-Series	ES, HW, SARIMA，SEIRD	Using the proposed optimized SEIRD, this study obtained a mean squared log error of 10^{-3} order.	While univariate models (Holt Winters', SARIMA) lead to highly accurate predictions, in addition to their simplicity of implementation and procedure and their ability to deal with time series seasonality and cycles, they are not as extendable as multivariate epidemiological models.
[12]	Forecast COVID-19 confirmed cases at the country level.	Machine & Deep Learning	LR, SVM, MLP, RF	BiLSTM performed well, especially in UAE (confirmed cases: RMSE = 0.023, MAE = 0.0176, R^2 = 0.97).	The DL models have demonstrated crucial enhancements when dealing with time-series data in various applications.
[13]	Predict the spread of COVID-19 in the Southern Africa.	Time-Series	ARIMA	The ARIMA (11, 1, 11) model achieved the lowest RMSE (1005.02).	The ARIMA (11, 1, 9) was the best candidate for the training set. A 15-day forecast was also made from the model, which shows a perfect fit with the testing set.
[14]	Examine nationwide modelling of COVID-19 incidence in the United States using machine-learning algorithms.	Machine Learning	LR, ANN	The MLP with one hidden layer (RMSE = 0.722, MAE = 0.36) outperformed linear regression model (RMSE = 0.99, MAE = 0.58), and MLP with two hidden layers (RSME = 0.84, MAE = 0.40).	The distribution of the COVID-19 incidence rate in the United States was clustered. Age-adjusted mortality rates of ischemic heart disease, pancreatic cancer, leukemia, Hodgkin's disease, mesothelioma, and cardiovascular disease were highly relevant to COVID-19 incidence rates.
[15]	Develop an intelligent computing model for forecasting the outbreak of COVID-19.	Time-Series	ARIMA	For confirmed cases, ARIMA (2, 2, 2) performed best (RMSE = 5260.5). For recovered cases, ARIMA (1, 1, 1) performed best (RMSE = 2169.5). For deceased cases, ARIMA (2, 1, 0) performed best (RMSE = 101.37).	The ARIMA model was robust in forecasting the future trend of confirmed cases of the pandemic.
[16]	Develop a novel multimodal approach that combines network-valued and spatio-temporal disease data in an interpretable manner.	Deep Learning	GNN	The GNN with zero-inflated Poisson distribution yielded the lowest MSE (3.93) at 32 week, MSE (4.5) at 38 week, MSE (17.74) at 44 week and MSE (15.05) at 47 week.	This study incorporated the network data to make a notable improvement over existing approaches.
[17]	Find a proper model to predict and forecast COVID-19 deaths in Pakistan.	Machine Learning	ARIMA, MLP	The MLP (MSE = 354.00, RMSE = 18.81, MAE = 10.31) outperformed ARIMA (MSE = 435.13, RMSE = 20.86, MAE = 11.45).	The MLP model with 20 hidden layers outperformed all other competing models for modeling and prediction purposes.
[18]	Present a comparative study of machine learning-driven methods for COVID-19 transmission forecasting.	Deep Learning	LR, SVM, GAN, LSTM-CNN, GAN-GRU	The LSTM-CNN achieved improved performance with a MAPE of 3.718%.	The hybrid models (LSTM-CNN and GAN-GRU) outperformed the non-hybrid models, with LSTM-CNN achieving the best forecasting performance.
[19]	Determine the best predictive models and investigate the impact of lockdown policy on the COVID-19 in the USA.	Time-Series	NB INGARCH, GLM, ARIMAX	The NB INGARCH fitted the observed incidence better than ARIMA (AIC = 2223.84).	The results showed a statistically significant relationship between the lockdown policy in the USA and incidence and death counts.
[20]	Develop a data-driven multi-horizon incidence forecasting model considering the inter-country variability in static socio-economic factors.	Deep Learning	Temporal Fusion Transformer	The TFT model had high prediction accuracy, with 80% of the countries having MAPEs below 0.3.	The inter-country infection-related predictions vary widely over spatiotemporal variability, and different socio-economic variables have different influences over this inter-country variability.
[21]	Build models that allow forecasting possible new infections accurately in order to face the pandemic in specific sociocultural contexts in the best possible way.	Bayesian Spatiotemporal Model	Score-Driven Models, State-Space Model	The score-driven model provided the most accurate forecast of COVID19 cases (Argentina: MAPE = 18.86, Brazil: MAPE = 19.96).	The forecasting performance of the score-driven model was superior to that of the nonlinear state-space model with unobserved components for all countries.
[22]	Develop a system to forecast the spatiotemporal distribution of COVID-19 in Brazil and in the State of Pernambuco.	Machine Learning	LM, SVM, MLP, RF	The linear regression presented the best prediction results: correlation coefficient > 0.99 and RMSE (%) < 4% for Pernambuco and around 5% for Brazil.	The use of machine learning proved to be very effective in forecasting the spatial distribution of the cumulative cases of COVID-19.
[23]	Predict the COVID-19 outbreak in Bangladesh using a deep learning model.	Machine & Deep Learning	LSTM, SVM, RF	LSTM outperformed than other algorithms with achieving lowest train (RMSE = 53.14), test (RMSE = 274.86), and all (RMSE = 65.83) accuracy.	LSTM could be a perfect fit for real-time analysis.
[24]	Develop an AIT-based predictive model by choosing suitable ANN procedures to forecast the prognostic factors for the rapid spread of COVID-19 in Iraq.	Machine Learning	RBF, Fuzzy Cognitive Map (FCM), NARX	Comparatively, the accuracy of NARX (R^2 = 0.91, Accuracy = 0.85) was higher than the accuracy of the RBF (R^2 = 0.86, Accuracy = 0.81) and FCM (R^2= 0.9, Accuracy = 0.84) designed ANNs in training data.	An AI model was developed throughout this study based on three ANNs and achieved 81.6% performance accuracy.
[25]	1. Identify which function adjusts the best to the infected population growth in Mexico. 2. Determine the feature importance of climate and mobility. 3. Compare the results of different modeling approaches.	Time-Series & Deep Learning	LM, GLM, Polynomial Regression, ARIMA, ES, VAR, LSTM	The Sigmoid curve most accurately represented the growth of the infected population in Mexico and trends of accumulated daily cases and fatalities. The LSTM model outperformed other models in predicting daily cases (RMSE = 275.4) and fatalities (RMSE = 31.9).	The logistic growth model best fitted the pandemic's behavior, that there was enough correlation between climate and mobility variables and disease numbers, and showed that the LSTM network can be exploited for predicting daily cases.
[26]	Verify the influence of particulate matter concentrations and meteorological factors on the spread of COVID-19.	Deep Learning	K-Means, LSTM	The LSTM prediction model performed best in predicting confirmed cases of COVID-19 (RMSE = 0.09, MAE = 0.09, MAPE = 0.42).	LSTM had better performance and accuracy in predicting the number of confirmed cases of COVID-19, based on particulate matter concentration data and meteorological factors.
[27]	Forecast the spread of COVID-19 infections among selected	Deep Learning	LSTM,BiLSTM,	The random split encoder-decoder LSTM model provides the best test	The univariate model outperformed the multivariate model in this study.

	states in India.		ED-LSTM	performance in comparison to the rest of the models (RMSE = 10732).	
[28]	Provide estimations about how the virus will migrate inside an area over time.	Deep Learning	CNN, LSTM, CNN-LSTM	Compared to other machine learning methods, this spatiotemporal CNN-LSTM achieved the lowest RMSE (1.93) in Madrid areas.	The proposed model could learn both spatiotemporal patterns based on a sequence of COVID-19 incidence maps, with the spatial pattern extraction and the time sequence analysis working together to optimize the estimation power of the model.
[29]	Develop a machine learning-based model to forecast the daily growth rate and mortality rate of COVID-19.	Machine Learning	RF, SHAP	The single-objective optimization was likely to return a larger reduction in the unwanted cases compared to multi-objective optimization.	The temperature and the stringency index were identified as the top two important features to exert a greater impact on virus transmission.
[30]	Discover spatio-temporal predictive epidemic models from mobility and infection data.	Deep Learning	ES, ARIMA, LSTM	The proposed approach, which combines density-based clustering method to detect epidemic hot spots and LSTM model to predict epidemic spread, largely achieves better performances than other algorithms in terms of MAE, MAPE, and MSE for all five zip codes.	The proposed approach based on mobility patterns has good performance in the epidemic prediction domain.
[31]	Study the spatial effects of infected cases of COVID-19 in Makkah, Jeddah, and Taif.	Time-Series	ARIMA,STARIMA	STARIMA models were more reliable in forecasting future epidemics of COVID-19 than ARIMA models (with lower MAE, MSE and RMSE).	The STARIMA model outperforms the univariate ARIMA model with the inclusion of spatial information.
[32]	Develop statistical techniques with high forecasting accuracy and reliability for COVID-19.	Ensemble Learning	EEMD-ARIMA-NARANN	The mixture model showed the lowest values of the measurement metrics (South Africa: MAPE = 1.17, Nigeria: MAPE = 1.388).	This novel data-driven hybrid model did a better job of capturing the dynamic, changing trends of the target data than the others used in this work.
[33]	Estimate the prevalence of COVID-19 in Italy, Spain, and France.	Time-Series	ARIMA	In Italy, ARIMA (0,2,1) performed best (MAE = 850.43, MAPE = 4.75). In Spain, ARIMA (1,2,1) performed best (MAE = 1147.89, MAPE = 6.68). In France, ARIMA (2,2,1) performed best (MAE = 635.87, MAPE = 6.25).	ARIMA models are suitable for predicting the prevalence of COVID-19 in the future.
[34]	Interpret the distribution of COVID-19 and develop an effective COVID-19 forecasting model in Egypt.	Time-Series	ARIMA, ES, MA, Quadratic/ Linear Trend Model	Compared to other time-series methods, the ARIMA (2,1,2) model achieved the lowest MAPE (12.3).	The total number of COVID-19 cases in mainland Egypt could reach 11076 per week (March 1, 2020 through January 24, 2021), and the number of simple regenerations could reach 12. Analysis of the ARIMA sequences shows a rise in the number of COVID-19 events.
[35]	Explore the potential utility of local COVID-19 infection incidence data in developing a forecasting model for the COVID-19 hospital census.	Machine Learning	Vector Error Correction Model (VECM)	The VECM (MAPE = 5.9%) achieved lower MAPE compared to ARIMA model (MAPE = 6.6%).	The multivariate VECM provided a very good fit to the data and outperforms models with no or other leading indicators.
[36]	Develop a deep learning approach to forecast the pandemic trend for Brazil, India, and Russia.	Deep Learning	CNN, LSTM, CNN-LSTM	The CNN-LSTM model had the highest performance in India (MAE = 5245) and Russia (MAE = 986), while LSTM model had the highest performance in Brazil (MAE = 15275).	All CNN, LSTM, and CNN-LSTM models successfully captured the transmission trend in each country. This study observed that the LSTM model has the best performance based on the results of evaluation metrics.
[37]	Verify if there is a relationship between forecast performance and data complexity.	Bayesian Spatio temporal Model	State-Space Model, LSTM	The state-space models outperformed LSTM models in Arabian Gulf countries except for Saudi Arabia (RMSE(LSTM) = 109.11, RMSE (state-space model) = 111.19).	The state space models outperform the LSTM to predict accurately in the presence of highly complex health surveillance data.
[38]	Identify the data-generating process of three COVID-19 variables (daily new infections, deaths, and recovered cases) using DLM to provide a forecast in Pakistan.	Bayesian Spatio temporal Model	Bayesian Dynamic Linear Model	The Bayesian dynamic linear model captured the variability of the data very well, with elegantly model a time series with non-stationarity nature, structural breaks, no clear pattern.	The forecast findings of the study indicated that the average daily number of new cases is higher than the average values of the observed data.
[39]	Understand the COVID-19 pandemic curve in Jakarta using ARIMA and Exponential Smoothing Methods.	Time-Series	ARIMA	ARIMA was the best fitting model because it has the highest R^2 (0.97) and the lowest MSE (43.64), and RMSE (208,190), compared to Holt's Linear and Holt-Winters' Additive models.	ARIMA could be the best model to forecast the upcoming number of infected cases of COVID-19 in Jakarta.
[40]	Estimate the epidemiological trends of COVID-19 prevalence and mortality using the advanced α-Sutte indicator.	Time-Series	ARIMA, α-Sutte Indicator	The α-Sutte Indicator (MAE = 10325.7) was found to produce lower forecasting error rates than the ARIMA model (MAE = 11335.76) in all data apart from the prevalence testing set globally.	The α-Sutte Indicator has priority over the ARIMA model for forecasting the epidemiological trends of the prevalence and mortality of the COVID-19 outbreak in the mentioned regions except for the epidemiological trends of the COVID-19 prevalence around the globe.
[41]	Observe and predict the epidemiology of COVID-19 in Bangladesh based on daily confirmed cases.	Time-Series	AR, MA, ARMA, ARIMA	ARIMA was the most appropriate time series model for predicting the number of confirmed COVID-19 cases (MAE = 1965, MAPE = 0.28).	The best forecasting model for predicting the daily confirmed cases' trend in Bangladesh was discovered to be ARIMA (8, 1, 7).
[42]	Estimate the prevalence trend in Ukraine, Romania, the Republic of Moldova, Serbia, Bulgaria, and Hungary as Central European countries.	Time-Series	ARIMA	Different countries had different best-fit ARIMA models.	Different ARIMA models were chosen for different countries as the best models, depending on their lowest MAPE values.
[43]	Estimate the number of confirmed cases globally by analysing historical data pertaining to the growth rate in the number of confirmed COVID-19 cases together with relevant social environment factors.	Deep Learning	Optimized Data Assimilated Neural Network (ODANN)	The proposed ODANN model achieved lowest RSME (0.00282) and MAE (0.00214).	Information from Twitter offers valuable insights into people's emotional responses towards COVID-19, which has been proven to be useful in maximizing the accuracy and performance of our predictive model when coupled with data assimilation at the selected hidden layer(s) of the deep learning model.
[44]	Develop three models for predicting the dynamics of the COVID-19 epidemic process in specific areas using statistical machine learning methods.	Machine Learning	RF, KNN, GBM	The GBM outperformed in South Korea (MAE = 27.33), Ukraine (MAE = 396.67), and Germany (MAE = 370.4) in forecasting cumulative new cases, while the KNN model performed best in Japan (MAE = 49.43).	All models showed sufficient accuracy in deciding to implement control measures to counter the COVID-19 pandemic.
[45]	Predict the number of COVID-19 infections in different countries in America.	Machine Learning	MLP, SVM	SVM performed better in Brazil (MAE = 2508), Colombia (MAE = 76), and Peru (MAE = 686), while MLP performed better in Mexico (MAE = 231), Chile (MAE = 504), and the USA (MAE = 799).	An MLP can predict better when an optimization algorithm to determine the hyperparameter (number of layers and number of neurons per layer) is applied. However, a global minimum was not found for all cases, making it necessary to use a SVM when MLP did not perform well.
[46]	Investigate and model the effect of climate on the transmission pattern.	Time-Series	ARIMA, SARIMA, ARIMAX, SARIMAX	In Hongkong, SARIMAX(0,1,2)(1,0,0) with LST, RF and RH achieved the lowest RMSE (0.37). In Marucopa, SARIMAX(0,1,0)(1,0,0) with Pmax achieved the lowest RMSE (0.54).	Include the climatic variables as input series, which results in models with better performance than the univariate model, where influenza cases depend only on their past values and error signals.
[47]	Model the COVID-19 outbreak with different time series models and also predict the indicators.	Time-Series	ARIMA, ES, HW	Six indicators, in varied time periods and diverse countries, exhibited a propensity for different optimal time-series models.	More accurate predictions for the future can be obtained using time series models with a wide range of data from different countries by modelling real-time and retrospective data.
[48]	Investigate the spread of COVID-19 by fitting a GWRM to new daily cases in Senegal and making short-term predictions.	Generalized Regression	GLM, GWRM	The GWRM fitted the data better compared to GLM, with lowest AIC (1829),BIC (1848.67),RMSE (29.93) and MAE (22.89).	The GWRM fitted better the data than most of the usual count data, such as the negative binomial regression model.
[49]	Model and predict the number of COVID-19 infections, drawing out the effects of its spatial diffusion.	Generalized Regression	SGLMM	The SGLMM model provided good forecasts for the number of infections at local level while controlling for delayed reporting.	Locally, the provinces first concerned by containment measures are those that are not affected by the effects of spatial neighbours. The component accounting for the spatial interaction with surrounding areas is prevalent in provinces that are strongly involved in contagions.
[50]	Show that relatively simple additional analyses of the residuals can improve the modelling.	Time-Series	AR, GP	After modelling residuals, the value of R^2 has increased to 83% from 42%, indicating a good model fit.	The squared residuals can reveal the presence of the remaining seasonal variation in the incidence of influenza A.
[51]	Predict daily COVID-19 cases in 10 African countries spread across the north, south, east, west, and central Africa.	Machine Learning	ANN, ANFIS, SVM, LM, ANN-Ensemble and SVM-Ensemble	The proposed ensemble approaches demonstrated very high improvements in predicting the COVID-19 pandemic in Africa (RMSE = 0.0002, RMSE = 0.0155, and R^2 = 0.96).	A solid foundation for the application of ensemble approaches for predicting the COVID-19 pandemic across all regions and countries in the world was presented.
[52]	Describe the dynamic of the pandemic across 35 European countries over a period of 9 months.	Simple Regression	HC, LM	The multiple regression model outperformed the simple linear regression model for prediction the threshold value.	The model showed strongly connected deaths and incidences during the waves in spring and fall.
[53]	Forecast the pandemic of COVID-19 using machine learning methods.	Machine & Deep Learning	SIR, SVM, Polynomial Regression, LSTM	LSTM was able to predict the results with greater accuracy and precision (Precision = 0.81, Recall = 0.41, F1-Score = 0.55).	The proposed LSTM model was the most efficient among the machine learning models deployed in the experiment. The polynomial regression was affected by outliers in the data, whereas SVM was affected by overfitting and underfitting of the data.
[54]	Analyze, model, and predict the future trend of the COVID-19 pandemic in Pakistan.	Machine Learning	RF, XGBoost, LM	The random forest and XGBoost algorithms provided better accuracy than linear regression (with higher precision, accuracy and F1-score).	The random forest and XGBoost algorithms provided better accuracy than linear regression in predicting COVID-19 cases in Pakistan.

[55]	Forecast future COVID-19 cases based on past infections. Predict current COVID-19 cases using PM2.5, temperature, and humidity data with four machine learning classifiers.	Machine Learning	DT, RF, SVM, KNN	Based on RMSE values, KNN and SVM algorithms were found to be the best for predicting future incidences of COVID-19 based on past histories.	Temperature was found to be the best predictor for the number of COVID-19 cases, followed by relative humidity.
[56]	Develop a time series model using Australian influenza surveillance and local internet search query data to predict seasonal influenza epidemics in the northern hemisphere.	Time-Series	SARIMA	The SARIMA model achieved low MAPE values in China (16.76), the US (96.97), and the UK (125.42).	The SARIMA model (1,1,1) (1,0,2), (2,2,2) (2,0,0), and (3,0,2) (1,0,0) with Australian infuenza and local search data was found to provide the best fit to the data in China, the US, and the UK, respectively.
[57]	Understand the behavior of the spread of COVID-19 as well as the projection of infections and deaths.	Time-Series	ARIMA, State-Space Model, GLARMA, ES, HW, Damped Trend Model	ARIMA stood out as the optimal time series model for forecasting confirmed COVID-19 case numbers (MAE = 454.27, MAPE = 17.54), while the damped trend method is best suited for predicting COVID-19-related deaths in Chile (MAE = 26.08, MAPE = 0.37).	The dataset used in this research indicated that the most appropriate model is the ARIMA time series model for predicting the number of confirmed COVID-19 cases, whereas for predicting the number of deaths from COVID-19 in Chile, the most suitable approach is the damped trend method.
[58]	Predict the active rate, the death rate, and the cured rate in India by analyzing the data from COVID-19.	Machine Learning	SVM, Prophet, LM	The Prophet model has been shown to be the best predictive method for predicting active rate (MAE = 31.55), death rate (MAE = 1.3) and cured rate (MAE = 52.12) compared to SVM and the linear regression.	In this study, the Prophet forecasting model was the best predictive method for predicting the active rate, death rate, and cure rate compared to SVM and linear regression in a dataset with vast uncertainty or small size.
[59]	Predict the number of COVID-19 positive cases in 36 states of Nigeria using the LSTM algorithm of deep learning.	Machine & Deep Learning	K-Means, PCA, LSTM, NB, XGBoost, SVM	LSTM was observed to be superior among all proposed algorithms with highest accuracy (98.1%).	The approaches of PCA and K-Means integration significantly enhanced the performance accuracy of various algorithms employed to model our dataset. LSTM had the greatest prediction accuracy of 98.1%.
[60]	Investigate and assess the effectiveness of preventive measures by the government of Egypt to control the spread of COVID-19.	Simple Regression	Polynomial Regression	The best models predicted accurately for the next 15 days (adjusted $R^2 >$ 0.9).	The exponential, fourth-degree, fifth-degree, and sixth-degree polynomial regression models are excellent models, especially the fourth-degree model, that will help the government prepare their procedures for one month.
[61]	Predict the trend of COVID-19 deaths in Pakistan using statistical models.	Simple Regression	LM, LR, Quadratic Regression	The quadratic model outperformed than linear model and logarithmic model (AIC = 330.71, BIC = 141.81).	This study stated that the rate of mortality would decrease by the end of October 2020.
[62]	Develop models that can be applied for real-time prediction of COVID-19 activity in all individual countries and territories worldwide.	Machine Learning	HC, RF	The RF model (MAE = 5.42) outperformed the DT (MAE = 6.78) and LSTM (MAE = 9.13) models. Adding the Google Trends data improved the performance of the models using incidence data (MAE = 5.67).	By integrating previous incidence and Google Trends data, this study's machine learning algorithm was able to predict the incidence of COVID-19 in most individual countries and territories accurately 7 days ahead.
[63]	Provide insight into how different models of machine learning are contrivances in the current situation.	Machine Learning	LM, SVM, RF, KNN, ES	ES performed best with the highest R^2(new infected: 0.99, death: 0.98) and the lowest RMSE (new infected: 25136.3, death: 3440.96).	In this study, different machine learning algorithms showed different results in different class predictions, and model performance improved by increasing the size of the training set.
[64]	Construct a web tool resource that provides forecasts of the case and death numbers of COVID-19 over the short-term future in Bangladesh.	Machine Learning	LM, Polynomial Regression, SVM, MLP, Polynomial-MLP, Prophet	Different models showed different forecasting abilities at different time periods. Prophet can be estimated as a more stable regression model than others.	The proposed approach can accurately forecast the number of infected cases daily by training the prior 25 days of sample data recorded on our web application.
[65]	Forecast COVID-19 on the basis of ANN, KF, LSTM, and SVM methods.	Machine & Deep Learning	EnKF, SVM, ANN, LSTM	The performance of LSTM (RMSE = 687.54) was better than that of the ANN (RMSE = 718.09), KF (RMSE = 746.98), and SVM (RMSE = 709) methods.	LSTM provided better forecasts almost in all cases as compared to Kalman Filter, ANN, and SVM for the short term.
[66]	Explore and compare predictive potential in the sense of cumulative weekly forecasting of COVID-19 cases in India using machine learning regression and statistical models.	Time-Series	ARIMA	The ftting values and predicted values of the ARIMA model reasonably matched the real incidence of the COVID-2019 diseases, as evaluated by MAE.	The west and south of the Indian district are highly vulnerable to COVID-19.
[67]	1. Study the impact of adding mobility on prediction performance. 2. Find out how using multiple sample time series affects the performance of the predictions.	Deep Learning	LSTM	The predicted (RMSE = 134.01) results had similar accuracy and spatial patterns with the ensemble model (RMSE = 175.64) used for benchmarking of CDC.	Results showed that adding mobility as a variable and using multiple samples to train the network improved predictive performance both in terms of bias and variance of the forecasts.
[68]	Conduct reliable daily forecasts and provide a long-term prevalence trajectory of the disease.	Time-Series	ARIMA	All ARIMA models achieved MAPE less than 4%.	In the coming four months, the number of COVID-19-positive people in Ethiopia may reach 56,610 from 5,846 on June 30, 2020, in the average rate scenario.
[69]	Draw an objective comparison of the performance of different time series models.	Time-Series & Deep Learning	MA, Linear/ Quadratic Trend Model, ES,ARIMA, LSTM, HW,SARIMAX, Sutte-ARIMA	Different time-series trends have different best-fitted models, evaluated by achieving the lowest RMSE and MAPE.	ARIMA models excelled in forecasting exponential growth. Holt's Linear Trend was best for mixed exponential-linear trends. The S-curve Trend model shined in logistic growth. Damped Trend models were top for damped trends. LSTM models suited stochastic trends. The Quadratic Trend model led in polynomial growth. Holt-Winters' additive model was perfect for cyclical increase-plateau trends. SARIMAX was superior for exponential trends with periodic damping. Sutte-ARIMA fit trends with constant levels and occasional spikes.
[70]	Estimate the epidemiological course of COVID-19 in Romania.	Time-Series	ARIMA	Different time periods had different best-fit ARIMA models.	Several ARIMA models were generated from March to August, and ARIMA (1,2,1) were considered the best models.
[71]	Forecast COVID-19 outbreaks in Bangladesh with higher accuracy.	Machine Learning	Prophet	The errors of cumulative total infections and cumulative total deaths were bounded by 10% and 5%.	This model predicted the daily and cumulative infection and death rates with a low error rate and predicted the different waves of outbreaks with higher accuracy.
[72]	Forecast the hospitalization of COVID-19 patients in Uttarakhand using available district Dehradun data and display the patterns of the disease for the next eight weeks.	Simple Regression	ES	In the forecasted model, the R^2value was 67% and the MAPE was approximately 10%, which indicated that the model was well fitted.	According to prediction, 5438 people will be subjected to hospitalization by September 2022, assuming that COVID cases will increase in the future and take on a lethal variety, as was the case with the second wave.
[73]	Construct models for predicting influenza incidence, thus providing research ideas for influenza prevention and aiding guidance for public health.	Time-Series	X-12-ARIMA	The X-12-ARIMA model fitted the data reasonably well, with low RMSE and high R^2.	The SARIMA (0,1,1) (0,1,1)12 forecasted the influenza incidence of Hebei, Guizhou, Henan, and Shandong Province; the SARIMA (1,0,0) (0,1,1)12 made forecasts in Gansu; the SARIMA (3,1,1) (0,1,1)12 made forecasts in Tianjin; and the SARIMA (0,1,1) (0,0,1)12 made forecasts in Hunan.
[74]	Analyze the historical data on the disease and use the collected data to forecast the trajectory of COVID-19 in order to construct robust and accurate models.	Machine Learning	ARIMA, Prophet	A small superiority of the ARIMA (for confirmed cases: MAE = 74.75, R^2 = 0.98) model was shown in terms of forecasting ability.	Both models were found to be accurate and robust in forecasting the time series of COVID-19 in Saudi Arabia for the considered period, with a small superiority of the ARIMA model in terms of forecasting ability and of Prophet in terms of simplicity and a few hyper-parameters.
[75]	Design a model particular to the children's hospital for short-term forecasts of H1N1 influenza incidence.	Simple Regression	Mathematical model	The proposed model, up to January 29, 2011, achieved a reduced RMSE of 1.62 for the 3-day forecasts for subsequent time points.	Ninety-five percent confidence intervals calculated around our model's forecasts were accurate to +-3.6 cases per 3-day period.

Abbreviations can be found in the supplementary material (Table S1)

Table S7. A list of published studies in spatiotemporal pattern analysis (n = 16)

Ref	Aims & Objectives	Methods	Algorithms & Models	Key Findings
[76]	Develop an advanced method to examine spatiotemporal trends in correlated counts and rates of COVID-19.	Bayesian Spatiotemporal Model	A Bayesian approach with an efficient MCMC posterior that relies on Gibbs Sampling	A dynamic impact of COVID-19 on socially vulnerable communities was found. At the outset of the pandemic, COVID-19 disproportionately impacted less vulnerable counties before spreading to more vulnerable areas.
[77]	Provide an insight into the rapid growth of confirmed COVID-19 cases in India.	Generalized Regression	GAM	Population and rural population played a major role in the spread of COVID-19.
[78]	Map the spatiotemporal pattern and forecast outbreaks of COVID-19.	Bayesian Spatiotemporal Model	Bayesian Spatiotemporal Model	The spatiotemporal distribution of COVID-19 was primarily temporal, while there was relatively little spatial interaction between the West Java counties.
[79]	Explore the spatial variability in the timing of RSV epidemics.	Bayesian Spatiotemporal Model	Harmonic Poisson Regression Model, Hierarchical Bayesian Spatial Model	Earlier epidemics were associated with larger household sizes and greater population density. Nearby localities had similar epidemic timing. RSV epidemics grew faster in areas with more local contact opportunities, and that epidemic spread followed a spatial diffusion process based on geographic proximity.
[80]	Analyze several aspects of the country-wise time profile evolutions of COVID-19.	Machine Learning	Dynamic Time Warping, K-Medoids Clustering	Significant differences and time profiles were found regarding both the registered cases and death time profiles for each country, leading to the identification of three distinctive clusters in the corresponding country time series with wide variability present in each cluster.
[81]	Analyze and contribute to the knowledge of the spatial patterns of COVID-19 at neighbourhood level from a space-time perspective.	Spatial Regression	The Nearest-Neighbour, Global Moran's I, Space-time 3D Bins, Emerging Hotspots, Linear Bivariate Analysis, Ordinary Least Squares	Non-random distributions of COVID-19 cases with cluster patterns were found.
[82]	Understand the temporal and spatial dynamics of COVID-19, and analyze and predict the COVID-19 epidemic in Italy.	Machine Learning	Global and Local Moran's I, Kulldorf's Space–time Scan Statistic, Kernel Density Estimation, Bayesian Network, RF, Adaboost, ANN, SVM	The Italian epidemic had a temporal trend and spatial aggregation. The epidemic was concentrated in northern Italy and gradually spread to other regions. The AUC of these six features (mask, pneumonia, thermometer, ISS, disinfection, and disposable gloves) was >0.9, indicating that these features had a large contribution to the prediction model.
[83]	1. Investigate the effectiveness of the practice of public health measures. 2. Assess the correlation between infections and deaths caused by COVID-19. 3. Explore the relationship between COVID-19 spread and geographical locations.	Functional Data Analysis	Functional PCA, Functional Canonical Correlations Analysis, EMCluster, Point and Interval Forecast	The practice of public health measures helped to reduce the growth rate of the epidemic outbreak over the nation. A high canonical correlation between confirmed and death cases has been observed. States that were geographically close to the hot spots were likely to be clustered together, and population density appeared to be a critical factor affecting the cluster structure.
[84]	Identify COVID-19 hot and cold spots of disease clustering and define the predictability performance of the Google relative search volume model in clustered and nonclustered areas of the USA.	Generalized Regression	Getis-Ord General G and Gi*, Poisson Regression	Huge clusters involving multiple states were found, which resulted from various control measures in each state. Variability in Google relative search volume model performance was found among states and time periods.
[85]	Identify the mathematical model and ecological determinants of COVID-19 infection and mortality across different countries during the first six months of the pandemic.	Simple Regression	Linear/ Exponential Regression	The number of COVID-19 new cases was significantly associated with mobility trends, temperature, humidity, and the proportion of patients aged 65 years or above. Similarly, COVID-19 mortality was significantly associated with mobility trends, temperature, humidity, and the PCI of countries.
[86]	Analyze the spatial characteristics of human infection with the H7N9 virus in China and assess the risk areas of the epidemic.	Machine Learning	Kernel Density Estimation, Standard Deviation Ellipse Analysis, Spatial and Temporal Scanning Cluster Analysis, MLP	The epidemic situation was significantly correlated with atmospheric pressure, temperature, and daily precipitation. Human infection with the H7N9 virus in China has obvious seasonality, and the high-risk areas for influenza epidemics were mainly in the Yangtze River Delta and Pearl River Delta and gradually spread to inland areas of China.
[87]	Compare the performance of the ANFIS and PCA-ANFIS models in modelling the spatial distribution of COVID-19 prevalence in the Golestan province.	Machine Learning	PCA, ANFIS, PCA-ANFIS	Combined with the PCA, the PCA-ANFIS accuracy significantly increased, compared to single ANFIS.
[88]	Investigate the distribution of COVID-19 in both space and time in Malawi.	Bayesian Spatiotemporal Model	Bayesian Mixed Effects Models using Integrated Nested Laplace Approximation (INLA)	The study found significant effects of location and time, with the two interacting. The spatial distribution of COVID-19 risk showed major cities being at greater risk than rural areas. A high proportion of elderly people was positively associated with COVID-19 risk, while poverty incidence was negatively associated with COVID-19 risk.
[89]	1. Assess the spatiotemporal patterns of COVID-19 spread. 2. Quantify temporal variations of the rate of infection. 3. Compare variations in the daily infection rate at the wilayat (county) level.	Spatial Analysis	Global Moran's I, Getis-Ord Gi*	COVID-19 had prominent regional properties in terms of geographical distribution among 61 wilayats, with significant spatiotemporal agglomeration.
[90]	Extend the concept of inferring infections and cases from deaths down to a local level while accounting for varying population characteristics, timing of first exposure, and other unexplained sources of spatial correlation.	Bayesian Spatiotemporal Model	Bayesian Mixed Effects Models using Integrated Nested Laplace Approximation (INLA)	The model, including temporally- and spatially-correlated random effects, was found to best accommodate the observed variation in COVID-19-related deaths after accounting for local population characteristics.
[91]	Explore the spatial pattern of the COVID-19 outbreak, including estimation of hotspots, clustering, spatial direction, and heterogeneity, with the help of GIS in different time frames.	Spatial Analysis	Global Moran's I,Getis–Ord Gi*, Inverse distance weighted, GWR, Average Nearest Neighbor, Standard Deviational Ellipses, Polynomial Regression	Positive spatial heterogeneity was found in COVID-19 cases in India.

Abbreviations can be found in the supplementary material (Table S1)

Table S8. A list of published studies in association analysis (n = 61)

Ref	Study Aim & Objectives	Methods	Algorithms & Models	Key Findings
[92]	Detect the climate factors that affect the spread of COVID-19 by using machine learning and deep learning techniques.	Machine & Deep Learning	SVM, LR, KNN, DT, RF, NB, Dense Convolution Network	The decrease in humidity, temperature, and wind speed aided in spreading COVID-19, but the increase in sunny hours increased the spread of COVID-19.
[93]	Explore the association between the COVID-19 transmission rates and meteorological parameters.	Machine Learning	GBM	The experimental results suggested that the GBM model can capture the correlation between the cases of COVID-19 and atmospheric parameters. A certain association between atmospheric factors and transmission rates of COVID-19 existed in some states of India.
[94]	Explore local characteristics that may make places more susceptible to the spatial spread of COVID-19.	Spatial Regression	GWR	Transmission risk rose with children, the elderly, population size, income, education, and open facilities during the pandemic. The spatial spread of the disease occurred by moving from well-developed to deprived neighbourhoods during the initial stages of the pandemic.
[95]	Investigate the influence of environmental factors (temperature, humidity, and sunshine) on the progression of the pandemic.	Spatial Regression	Spatial Seemingly Unrelated Regression Equations Model	Higher incidence was associated with higher GDP per capita, the presence of mass transit systems in the province, and more sunshine; in contrast, population density, the percentage of older adults, temperature, and humidity displayed negative associations with the incidence of COVID-19.
[96]	Evaluate the effect of the lockdown policy on daily reports of new cases across the lockdown stages.	Simple Regression	Segmented Regression Model	There was a significant reduction in the rate of increase in new COVID-19 cases during Lockdown 1.0 and then Lockdown 4.0, with no significant rebound increase in this rate during the subsequent easing of the lockdown policy.
[97]	Study the impact of the lockdown schedule on pandemic prevention and control in Ningbo, China.	Machine Learning	RF, KNN	Taking an appropriate and early lockdown schedule can minimise the number of COVID-19 confirmed cases.
[98]	Visualize the spatial-temporal epidemic information of COVID-19 and identify the factors important to the spread of COVID-19.	Spatial Regression	Mixed GWR, XGBoost, SHAP	The confirmed COVID-19 cases at the city level in China were significantly influenced by mobility, urban vibrancy, and the proportion of the aging population. The order of importance of the three influential factors was as follows: Wuhan migration index > the proportion of the elderly population > intracity vibrancy.
[99]	Analyze the effectiveness of national- and region-level quarantines in Chile during the pandemic.	Bayesian Spatiotemporal Model	State-Space Model	In 75% of the Chilean regions under study (12 regions out of 16), an effective Phase 1 quarantine, which was implemented to control and reduce the number of cases of COVID-19 infection, was observed. Regarding Phase 2, the COVID-19 pandemic was effectively managed in 31% (5 out of 16) of the regions.
[100]	Investigate the relationship between exposure to pollutants (local SO2, NO2, and PM10) and COVID-19 (cases and deaths) in Mumba.	Spatial Regression	Spatial Lag Model, Spatial Error Model	COVID-19 death in Mumbai was distinctly associated with higher exposure to NO2, population density, and the number of wastewater drains.
[101]	Explore the association between short-term exposure to air pollutants and COVID-19-confirmed cases.	Ensemble Learning	GAM, Ensemble-based Dynamic Emission Model, SHAP	Short-term exposure to ground-level ozone was positively related to daily COVID-19 confirmed cases; however, exposure to particulates, PM 2.5 and PM 10, depicted a negative association. Increased emissions from other counties positively affected COVID-19 cases in adjoining counties as well.
[102]	Investigate the association between COVID-19 and meteorological parameters in 188 countries by using statistical approaches.	Generalized Regression	GAM, Distributed Lag Nonlinear Model	The mean temperature, wind speed, and relative humidity were negatively correlated with daily new cases of COVID-19, and the diurnal temperature range was positively correlated with daily new cases of COVID-19.
[103]	Evaluate and quantify the associations between RSV activity and pneumococcal disease in infants.	Generalized Regression	Generalized Linear Model, Harmonic Regression Model	RSV and pneumococcal pneumonia shared a distinctive spatiotemporal pattern. RSV was associated with a significant increase in the incidence of pneumococcal pneumonia in children aged < 2 years. Influenza was also associated with an increase in pneumococcal pneumonia among children aged 1-2.
[104]	1. Examine the association of absolute humidity with the number of influenza cases and the strength of associations. 2. Examine how adjustments for seasonality and autocorrelation affected the results.	Distributed Lag Nonlinear Model	Distributed Lag Nonlinear Model	All models used in the study showed a significant increase in the number of influenza cases as absolute humidity decreased, although the magnitude of the association differed substantially by model. Furthermore, relative risk reached a peak at lag 10–14 with extremely low absolute humidity.
[105]	Understand the effects of meteorological factors on the prevalence and seasonality of common respiratory viruses in China.	Time-Series	ARIMA, Poisson GAM	RSV-associated hospitalizations were predictable, and the monthly number of RSV cases decreased by 11.25% for every 1-degree increase in the average temperature. The seasonality of certain respiratory viruses may be explained by meteorological influences.
[106]	Examine if BN learning algorithms can conceptualize the complex interaction of factors related to COVID-19 transmission.	Bayesian Spatiotemporal Model	Bayesian Network (BN) Model	All the BN models performed very well in predicting the COVID-19 cases as evidenced by low log loss and high recall values. COVID-19 was spreading, driven by increasing human interaction through increased mobility and shopping.
[107]	Measure the impact of climate characteristics on the prevalence rate of COVID-19 in Brazilian states, given the exogenous nature of these variables.	Spatial Regression Model	Spatial Durbin Model	An increase of 1% in the solar incidence, average temperature, and relative humidity of the air reduced COVID-19 prevalence rates by 0.16%, 0.049%, and 0.22%, respectively, considering the 11-day moving average.
[108]	Examine the community-level associations between exposure to ambient PM2.5 and incident influenza in Guangzhou, China.	Bayesian Spatiotemporal Model	Hierarchical Bayesian Spatiotemporal Model	Short-term exposures to PM2.5 over a 6-day lag of days 0–5 were associated with a higher incidence of influenza.
[109]	Explore the relationship between the incidence of influenza and climate under different climate conditions.	Time-Series	ARIMA	Relative humidity was associated with the incidence of influenza A in Singapore, Hong Kong, Brisbane, and Vancouver. In the case of influenza B, the mean temperature was the key climate variable associated with the incidence of influenza in Hong Kong, Brisbane, Melbourne, and Vancouver. Rainfall was not significantly correlated with the incidence of influenza A or B in any of these cities.
[110]	Understand the relationship between weather variables and the epidemic spreading of COVID-19 in Pakistan.	Machine & Deep Learning	ARIMA, LM, SVM, MLP, RNN, GRU, LSTM	Weather parameters hold more influence in evaluating the cases and deaths than community, age, and the total population. Temperature and humidity are salient parameters for predicting COVID-19-affected instances. The higher the temperature, the lesser the mortality due to the COVID-19 infection.
[111]	Examine the associations of daily average temperature and relative humidity with the daily counts of COVID-19 cases in 30 Chinese provinces.	Generalized Regression	GAM	Both average daily temperature and average relative humidity influenced the occurrence of COVID-19 in Hubei province and some other provinces, with the existence of spatial heterogeneity among provinces in Mainland China.
[112]	Quantify the contribution of the sales of hand hygiene products and the number of international and domestic airline passenger arrivals on the HRSV epidemic in Japan.	Generalized Regression	Generalized Linear Gamma Regression Model	The average number of monthly HRSV case notifications in 2020 decreased by approximately 85% compared to those in the preceding 6 years (2014–2019).
[113]	Explore how changes in human mobility shaped the transmission dynamics of COVID-19 during its first wave in the United States.	Bayesian Spatiotemporal Model	Bayesian Spatiotemporal Generalized Additive Mixed Model combined with Distributed Lag Model (DLM)	An increase in movement from home to workplaces, retail and recreation stores, public transit, grocery stores, and pharmacies 7 weeks ago was associated with an increase in the COVID-19 incidence rate.
[114]	Examine the effect of socio-ecological factors on the transmission of H1N1 in Brisbane, Australia.	Bayesian Spatiotemporal Model	Bayesian Spatialtemporal Conditional Autoregressive (CAR) Model	No significant association between socio-economic indexes for areas and H1N1 was observed. Average weekly temperature at a lag of one week and rainfall at a lag of one week were substantially associated with H1N1 incidence. The auto-regression term was significantly associated with H1N1 transmission.
[115]	Assess the effectiveness of social distancing measures on COVID-19 incidence and mortality in Iran.	Simple Regression	Segmented Regression Model	Social distancing significantly reduced the incidence and mortality of COVID-19 in Iran.
[116]	Explore the differences in the associations between weather variability and seasonal influenza and the growth rates of seasonal influenza epidemics among different age groups in Queensland, Australia.	Bayesian Spatiotemporal Model	Bayesian Spatiotemporal Model	Weather factors were significantly associated with seasonal influenza, with effects that differed by age group. A positive relationship between seasonal influenza and the Relative SocioEconomic Advantage and Disadvantage Index was also present in all three age groups.
[117]	Assess the effectiveness of social distancing on COVID-19 hospitalizations in a patient population in New York City.	Simple Regression	Segmented Regression Model, ARIMA	There was a significant decrease in the upward daily trend in the mean number of COVID-19 admissions and patients on ventilators after the assumed effective date of the New York State on PAUSE mandate.
[118]	Identify the most critical factors that are responsible for the overall fatalities caused by COVID-19 by using ML models.	Machine Learning	RF, GBM, SHAP	This study has identified a few causal factors that have exhibited a high association with COVID-19 counts across continents. Air pollution, migration, economy, and demographic factors were found to be the most significant controlling factors.
[119]	Explore the associated risk factors of the COVID-19 Delta variant.	Bayesian Spatiotemporal Model	Bayesian Spatiotemporal Model with Mixed Random Effects	For the COVID-19 Delta variant cases, the eight considered environmental, sociodemographic, and public intervention factors were selected in different clusters, and the factor effects had distinct temporal behaviours depending on the groups.
[120]	Understand the factors associated with the heterogeneity of in-hospital COVID-19 morbidity and mortality across France.	Generalized Regression	Negative-binomial GAM	Spatial heterogeneity existed in the in-hospital COVID-19 incidence and mortality rates following the spread of the epidemic. The delay between the first COVID-19-associated death and the onset of the national lockdown was positively associated with in-hospital incidence, mortality, and case fatality rates.
[121]	Identify the best GIS-based model that can explore, quantify, and model the determinants of COVID-19 incidence and fatality.	Spatial Regression	GWR	The main predictors of the COVID-19 incidence rate were overcrowding, health expenditure, HIV infections, air pollution, and BCG vaccination. The main determinants of COVID-19 fatality were the prevalence of bronchial asthma, tobacco use, poverty, aging, and cardiovascular disease fatality.

[122]	Understand the data-driven association and prediction of GI symptoms during the COVID-19 outbreak.	Generalized Regression	Negative-binomial GAM	GI symptoms, including diarrhea and loss of taste, would be good indicators for surveillance of SARS-CoV-2 infection and helpful for the early prediction of COVID-19 outbreaks for up to three weeks.
[123]	Examine the impact that economic, demographic, and mobility-related factors have had on the transmission of COVID-19 in 2020.	Bayesian Spatiotemporal Model	Hierachical Bayesian Poisson Generalized Linear Model	The spatial interrelationships between counties were a fundamentally important aspect of modelling the effect of mobility on the spread of COVID-19.
[124]	1. Investigate the effectiveness of the smart travel ban policy in Iran since November 21, 2020. 2. Examine the association between intercity travel patterns and COVID-19 trends in Iran.	Time-Series	ARIMA, ARIMAX	The interrupted time series analysis indicated that the smart travel ban policy had decreased intercity travel by around 29%. Moreover, the weekly intercity traffic increases the new weekly COVID-19 cases and deaths with a time lag of two and five weeks, respectively.
[125]	Investigate the effect of weather and socioeconomic and demographic factors on COVID-19.	Bayesian Spatiotemporal Model	Hierarchical Bayesian Spatiotemporal Model	The average land surface temperature was positively and higher education was negatively associated with the COVID-19 incidence.
[126]	Examine the associations between daily average temperature and relative humidity and the percent increase in COVID-19 cases.	Simple Regression	Fixed and random effects models with robust standard errors	Mean temperature, mean relative humidity, and malaria endemicity might have an essential role in the stability and transmissibility of the 2019 novel coronavirus.
[127]	Analyze the short-term effect of air pollution on COVID-19 transmission using county-level data collected during the 2020 wildfire season.	Generalized Regression	GAM	PM2.5, CO, and AQI were all significantly and positively associated with confirmed COVID-19 cases in all the moving average lags, while a negative relationship between NO2 and COVID-19 cases.
[128]	Unravel the dynamics of COVID-19 spread in the urban environmental context at finer resolutions at neighbourhood level through spatial analysis.	Spatial Regression	GWR	During the early phase of the COVID-19 epidemic in Singapore, a significant but weak correlation of temperature with COVID-19 incidence was observed in several sub-zones of Singapore. Across sub-zones, high residential population density and urbanization were associated with COVID-19 incidence.
[129]	Investigate the association between COVID-19 cases and two components, average temperature and relative humidity, in the 16 states of Germany.	Simple Regression	Panel Regression Model	The daily COVID-19 cases correlate negatively with the average temperature and positively with the average relative humidity.
[130]	Evaluate the lagged meteorological impacts on COVID-19 incidence by considering a long study period and diversified high-risk areas in the US.	Distributed Lag Nonlinear Model	Distributed Lag Nonlinear Model	Maximum temperature, minimum relative humidity, and precipitation better explained COVID-19 incidence than the other meteorological factors.
[131]	Explore the relationship between meteorological factors and RSV infections among hospitalized children using different statistical models.	Machine Learning	ARIMA, GAM, LASSO	The ARIMA model revealed a marked seasonal pattern in the RSV detection rate, which peaked in winter and spring. A lower temperature and higher wind speed increased RSV detection, while temperature and relative humidity were negatively correlated with RSV detection.
[132]	Explore whether meteorological factors' effects showed seasonal characteristics and spatial variations related to the macroclimate.	Simple Regression	Two-way Fixed Effect Regression Model, Kernel Density Estimation	The daily average temperature, humidity, and wind speed negatively affected the daily new cases, with humidity and temperature playing a dominant role. Spatially, the negative effects of temperature and humidity on COVID-19 transmission clustered in the northeastern and central parts of Brazil.
[133]	1. Investigate the spatio-temporal trend of COVID-19 incidence. 2. Investigate the association between deprivation index, population density, and COVID-19 cases while accounting for spatial and temporal correlation.	Bayesian Spatiotemporal Model	Hierarchical Bayesian Spatiotemporal Model	This study found an association between deprivation index and COVID-19 incidence for the most deprived quantile compared to the least deprived. There was a large range of spatial heterogeneity in COVID-19 cases in Ireland.
[134]	Understand the spatial relationships between the COVID-19 infection rate and key variables of air pollution, geo-meteorological, and social parameters in Dhaka, Bangladesh.	Spatial Regression Model	GWR	Out of 7 air pollution parameters, PM2.5, AOT, CO, water vapor, and O3 were highly correlated with the COVID-19 infection rate in this study. In geometeorological parameters, DEM, wind pressure, LST, rainfall, and wind speed were significantly associated with the COVID-19 infection rate. In social parameters, population density, brickfield density, and poverty had high coefficients for the COVID-19 infection rate.
[135]	Identify the global transmission trend of COVID-19 from the perspective of spatial correlation and spatial lag.	Spatial Regression Model	Spatial Lag Model, Spatial Error Model, Spatial Durbin Model	Concerning the COVID-19 patient series, there existed a spatial correlation between the 14 typical countries. COVID-19 patients can infect others with lag. There were relationships among the new COVID-19 patients, confirmed patients, rehabilitated patients, and deaths caused by COVID-19.
[136]	Understand whether climate factors influence the seasonality of RSV in Thailand.	Time-Series	ARIMA, ARIMAX	RSV activity correlated positively with rainfall and relative humidity but negatively with mean temperature.
[137]	Explore the association between air pollution and influenza cases in Jinan City, especially during the outbreak of COVID-19.	Generalized Regression	Poisson GAM	PM2.5, SO2, CO, and NO2 were significantly associated with the risk of influenza during 2020–2021. Influenza cases aged over 59 years had a slightly larger relative risk when exposed to all air pollutants (except O3) than the younger group.
[138]	Understand the short-term effect of air pollution on influenza.	Generalized Regression	GAM	Exposure to SO2, NO2, and O3 significantly increased the incidence of influenza in both the single- and multiday lag models.
[139]	Analyze the spatial distribution characteristics of COVID-19 cases and their relationships with meteorological and environmental factors.	Spatial Regression	GWR	There was no obvious spatial clustering of confirmed COVID-19 cases in Hubei province, while the decline and end of the newly confirmed cases revealed relatively obvious negative spatial correlations. The impacts of environmental and meteorological factors on the development of COVID-19 were insignificant.
[140]	Explore how the spatial and temporal patterns of COVID-19 disease across Portugal relate to the physical and human attributes of the territory at a higher temporal resolution (15 days).	Generalized Regression	Generalized Linear Mixed Model	There was a higher incidence of COVID-19 in municipalities, with a higher percentage of people working in the tertiary sector. A positive relationship was identified between COVID-19 incidence and air pollution. Municipalities where the average household size was higher were found to have significantly higher cases.
[141]	Identify the potential factors contributing to the COVID-19 incidence rate at the provincial level in Canada.	Spatial Regression	Spatial Lag Model, Spatial Error Model	Median income, diabetes, and unemployment significantly affected the COVID-19 rates in Canada.
[142]	Explore long-term exposure to weather conditions and the role of air pollution on the infection spread and mortality due to COVID-19 in India.	Bayesian Spatiotemporal Model	Bayesian Linear Regression	The spatial shifting of COVID-19 cases from the western to the southern and then eastern parts of India was well observed. More CFR was correlated with higher aerosol optical depth, O3, and NO2 in India.
[143]	Examine the relative significance of potential explanatory variables (n = 75) concerning COVID-19 prevalence and mortality using multilayer perceptron artificial neural network topology.	Machine Learning	ANN	Regarding COVID-19 prevalence, unemployment and population density were among the most influential variables. For COVID-19 mortality, health-related variables such as diabetes prevalence and the number of hospital beds were among the most significant variables.
[144]	Reveal the factors affecting the spread of the COVID-19 pandemic by applying spatial analysis tools.	Spatial Regression	Spatial Lag Model	There were spatial associations and distinct spatial clusters in COVID-19 cases at the provincial level in Turkey. Population density and elderly dependency ratio were very important in explaining the model of COVID-19 case numbers. COVID-19 was affected by the case numbers of neighboring provinces, apart from the said explanatory variables.
[145]	Quantify associations between temporal variations in COVID-19 incidence and meteorological variables globally.	Distributed Lag Nonlinear Model	Distributed Lag Nonlinear Model, Meta-Analysis	Globally, low temperatures and low absolute humidity were associated with higher COVID-19 incidences. There was substantial heterogeneity in the associations between the respective environmental exposures and COVID-19 risk between countries.
[146]	Present a data-driven approach for exploring the spatio-temporal patterns of the pandemic over a regional scale and a country scale, as well as what geographical variables could potentially contribute to expediting its spread.	Spatial Regression	GWR, RF, LASSO, SVM	Population density, amenities such as cafes and bars, and pollution levels were the most influential explanatory variables, while pollution levels can be explicitly used to monitor lockdown measures and infection rates at the country level.
[147]	Quantify the impact of the lockdown on the incidence of COVID-19.	Simple Regression	Ordinary Least Squares	There was a significant decrease in the COVID-19 daily cases reported in China following the institution of a lockdown.
[148]	Investigate the associations between meteorological factors and the daily number of new cases of COVID-19 in nine Asian cities.	Generalized Regression	GAM	Increased temperature yield increases the daily number of new cases of COVID-19.
[149]	Investigate the spatial clustering pattern of COVID-19 in mainland China at the prefecture level and explore the relevant factors influencing COVID-19.	Spatial Regression	Kulldorff's Space-time Scan Statistic, GWR, LASSO	Population outflow from Wuhan City significantly increased the risk of COVID-19. Relative humidity, precipitation, and NO2 were positively associated with COVID-19 incidence. Average wind speed and O3 were negatively associated with COVID-19 incidence.
[150]	Explore the correlation between climatic factors and the morbidity of COVID-19 in Wuhan, China, using a geographic detector and GWR model.	Spatial Regression	GWR	Wind speed, relative humidity, temperature, and air pressure were important meteorological factors affecting the spread of COVID-19 in Wuhan.
[151]	Investigate associations between tropical influenza incidence and weather variability among children under five in a poor urban area of Dhaka, Bangladesh.	Generalized Regression	Zero-inflated Poisson and Generalized Linear Poisson Model	Influenza A had associations with minimum temperature, relative humidity, sunlight duration, and rainfall, whereas only relative humidity was associated with influenza B.
[152]	Explore the spatial and temporal variation of H7N9 infection and assess	Bayesian	Bayesian Spatialtemporal	High spatial variations in the H7N9 risk were mainly observed in the east and centre of Shanghai municipality. The H7N9 incidence rate was significantly

	the effects of temperature and rainfall on H7N9 incidence.	Spatiotemporal Model	Conditional Autoregressive (CAR) Model	associated with the fortnightly mean temperature.

Abbreviations can be found in the supplementary material (Table S1)

Table S9. Quality scores for assessing the risk of bias in the included articles (n = 152)

Ref	Quality Assessment										
	Did the study address a clearly focused issue?	Did the authors use an appropriate method to answer their question?	Were the measures accurately measured to reduce bias?	Were the data collected in a way that addressed the research issue?	Did the study have enough data to minimise the play of chance?	How are the results presented?	Was the data analysis sufficiently rigorous?	Is there a clear statement of findings?	Can the results be applied to the other settings?	How valuable is the research?	Total score
[1]	1	1	1	1	1	1	1	1	0	1	9
[2]	1	1	1	1	0	1	0	1	1	1	8
[3]	1	1	1	1	1	1	1	1	1	1	10
[4]	1	1	1	1	0	1	1	1	0	1	8
[5]	1	1	1	1	0	1	1	1	0	1	8
[6]	1	1	1	1	1	1	1	1	0	1	9
[7]	1	1	1	1	0	1	1	1	0	1	8
[8]	1	1	1	1	0	1	1	1	0	1	8
[9]	1	1	1	1	1	1	1	1	0	1	9
[10]	1	1	1	1	1	1	1	1	1	1	10
[11]	1	1	1	1	1	1	1	1	1	1	10
[12]	1	1	1	1	1	1	1	1	1	1	10
[13]	1	1	1	1	1	1	1	1	0	1	9
[14]	1	1	1	1	0	1	1	1	0	1	8
[15]	1	1	1	1	1	1	1	1	1	1	10
[16]	1	1	1	1	0	1	1	1	0	1	8
[17]	1	1	1	1	1	1	1	1	0	1	9
[18]	1	1	1	1	0	1	1	1	1	1	9
[19]	1	1	1	1	0	1	1	1	0	1	8
[20]	1	1	0	1	0	1	1	1	1	1	8
[21]	1	1	1	1	1	1	1	1	1	1	10
[22]	1	1	1	1	0	1	1	1	0	1	8
[23]	1	1	1	1	0	1	1	1	0	1	8
[24]	1	1	1	1	0	1	1	1	0	1	8
[25]	1	1	1	1	0	1	1	1	1	1	9
[26]	1	1	1	1	0	1	1	1	0	1	8
[27]	1	1	1	1	0	1	1	1	0	1	8
[28]	1	1	1	1	1	1	1	1	1	1	10
[29]	1	1	1	1	0	1	1	1	1	1	9
[30]	1	1	1	1	0	1	1	1	0	1	8
[31]	1	1	1	1	0	1	1	1	0	1	8
[32]	1	1	1	1	0	1	1	1	1	1	9
[33]	1	1	1	1	0	1	0	1	1	1	8
[34]	1	1	1	1	0	1	1	1	0	1	8
[35]	1	1	1	1	0	1	1	1	0	1	8
[36]	1	1	1	1	0	1	1	1	1	1	9
[37]	1	1	1	1	1	1	1	1	1	1	10
[38]	1	1	1	1	1	1	1	1	0	1	9
[39]	1	1	1	1	0	1	1	1	0	1	8
[40]	1	1	1	1	0	1	1	1	1	1	9
[41]	1	1	1	1	0	1	1	1	0	1	8
[42]	1	1	1	1	0	1	1	1	1	1	9
[43]	1	1	1	1	0	1	1	1	1	1	9

[44]	1	1	1	1	1	1	1	1	1	1	10
[45]	1	1	1	1	1	1	1	1	1	1	10
[46]	1	1	1	1	1	1	1	1	1	1	10
[47]	1	1	1	1	1	1	1	1	1	1	10
[48]	1	1	1	1	0	1	1	1	0	1	8
[49]	1	1	1	1	0	1	1	1	0	1	8
[50]	1	1	1	1	1	1	0	1	0	1	8
[51]	1	1	1	1	1	1	1	1	1	1	10
[52]	1	1	1	1	0	1	1	1	1	1	9
[53]	1	1	1	1	0	1	1	1	1	1	9
[54]	1	1	1	1	1	1	1	1	0	1	9
[55]	1	1	1	1	0	1	1	1	0	1	8
[56]	1	1	1	1	1	1	1	1	1	1	10
[57]	1	1	1	1	0	1	1	1	0	1	8
[58]	1	1	1	1	0	1	1	1	0	1	8
[59]	1	1	1	1	0	1	1	1	0	1	8
[60]	1	1	1	1	0	1	1	1	0	1	8
[61]	1	1	1	1	0	1	1	1	0	1	8
[62]	1	1	1	1	0	1	1	1	1	1	9
[63]	1	1	1	1	0	1	1	1	0	1	8
[64]	1	1	1	1	0	1	1	1	0	1	8
[65]	1	1	1	1	1	1	1	1	0	1	9
[66]	1	1	1	1	0	1	1	1	0	1	8
[67]	1	1	1	1	1	1	1	1	0	1	9
[68]	1	1	1	1	0	1	1	1	1	1	9
[69]	1	1	1	1	0	1	1	1	1	1	9
[70]	1	1	1	1	0	1	1	1	0	1	8
[71]	1	1	0	1	0	1	0	1	0	1	6
[72]	1	1	1	1	1	1	1	1	0	1	9
[73]	1	1	1	1	1	1	1	1	0	1	9
[74]	1	1	1	1	1	1	1	1	0	1	9
[75]	1	1	0	1	1	1	1	1	0	1	8
[76]	1	1	1	1	0	1	1	1	0	1	8
[77]	1	1	1	1	0	1	1	1	0	1	8
[78]	1	1	1	1	0	1	1	1	0	1	8
[79]	1	1	1	1	1	1	1	1	0	1	9
[80]	1	1	1	1	1	1	1	1	1	1	10
[81]	1	1	1	1	1	1	1	1	0	1	9
[82]	1	1	1	1	0	1	1	1	0	1	8
[83]	1	1	1	1	0	1	1	1	0	1	8
[84]	1	1	1	1	0	1	1	1	0	1	8
[85]	1	1	1	1	0	1	0	1	1	1	8
[86]	1	1	1	1	1	1	1	1	0	1	9
[87]	1	1	1	1	1	1	1	1	0	1	9
[88]	1	1	1	1	0	1	1	1	0	1	8
[89]	1	1	1	1	0	1	1	1	0	1	8
[90]	1	1	1	1	0	1	1	1	0	1	8
[91]	1	1	1	1	0	1	1	1	0	1	8

[92]	1	1	1	1	0	1	1	1	1	1	9
[93]	1	1	1	1	1	1	1	1	0	1	9
[94]	1	1	1	1	0	1	1	1	0	1	8
[95]	1	1	1	1	0	1	1	1	0	1	8
[96]	1	1	1	1	0	1	1	1	0	1	8
[97]	1	1	1	1	0	1	1	1	0	1	8
[98]	1	1	1	1	0	1	1	1	0	1	8
[99]	1	1	0	1	1	1	1	1	0	1	8
[100]	1	1	1	1	0	1	1	1	0	1	8
[101]	1	1	1	1	0	1	1	1	0	1	8
[102]	1	1	1	1	1	1	1	1	1	1	10
[103]	1	1	1	1	1	1	1	1	0	1	9
[104]	1	1	1	1	1	1	1	1	0	1	9
[105]	1	1	1	1	1	1	1	1	0	1	9
[106]	1	1	1	1	0	1	1	1	0	1	8
[107]	1	1	1	1	0	1	1	1	0	1	8
[108]	1	1	1	1	1	1	1	1	0	1	9
[109]	1	1	1	1	1	1	1	1	1	1	10
[110]	1	1	1	1	0	1	1	1	0	1	8
[111]	1	1	1	1	0	1	1	1	0	1	8
[112]	1	1	1	1	1	1	1	1	0	1	9
[113]	1	1	1	1	0	1	1	1	0	1	8
[114]	1	1	0	1	1	1	0	1	0	1	7
[115]	1	1	0	1	0	1	0	1	0	1	6
[116]	1	1	1	1	0	1	1	1	0	1	8
[117]	1	1	0	1	0	1	0	1	0	1	6
[118]	1	1	1	1	0	1	1	1	1	1	9
[119]	1	1	1	1	0	1	1	1	0	1	8
[120]	1	1	1	1	0	1	1	1	0	1	8
[121]	1	1	1	1	0	1	1	1	1	1	9
[122]	1	1	0	1	0	1	1	1	1	1	8
[123]	1	1	1	1	0	1	1	1	0	1	8
[124]	1	1	1	1	1	1	1	1	0	1	9
[125]	1	1	1	1	1	1	1	1	0	1	9
[126]	1	1	1	1	0	1	1	1	0	1	8
[127]	1	1	1	1	1	1	1	1	0	1	9
[128]	1	1	1	1	0	1	1	1	0	1	8
[129]	1	1	1	1	0	1	1	1	0	1	8
[130]	1	1	0	1	0	1	1	1	0	1	7
[131]	1	1	1	1	1	1	1	1	0	1	9
[132]	1	1	1	1	0	1	1	1	0	1	8
[133]	1	1	1	1	0	1	1	1	0	1	8
[134]	1	1	1	1	0	1	1	1	0	1	8
[135]	1	1	1	1	0	1	1	1	1	1	9
[136]	1	1	1	1	1	1	1	1	0	1	9
[137]	1	1	1	1	1	1	1	1	0	1	9
[138]	1	1	1	1	1	1	1	1	0	1	9
[139]	1	1	1	1	0	1	1	1	0	1	8

[140]	1	1	1	1	0	1	1	1	0	1	8
[141]	1	1	1	1	1	1	1	1	0	1	9
[142]	1	1	1	1	1	1	1	1	0	1	9
[143]	1	1	1	1	1	1	1	1	1	1	10
[144]	1	1	1	1	0	1	1	1	1	0	8
[145]	1	1	1	1	0	1	1	1	1	1	9
[146]	1	1	1	1	0	1	1	1	0	1	8
[147]	1	1	1	1	0	1	1	1	0	1	8
[148]	1	1	1	1	0	1	1	1	1	1	9
[149]	1	1	1	1	0	1	1	1	0	1	8
[150]	1	1	1	1	0	1	1	1	0	1	8
[151]	1	1	1	1	1	1	0	1	0	1	8
[152]	1	1	1	1	0	1	1	1	0	1	8

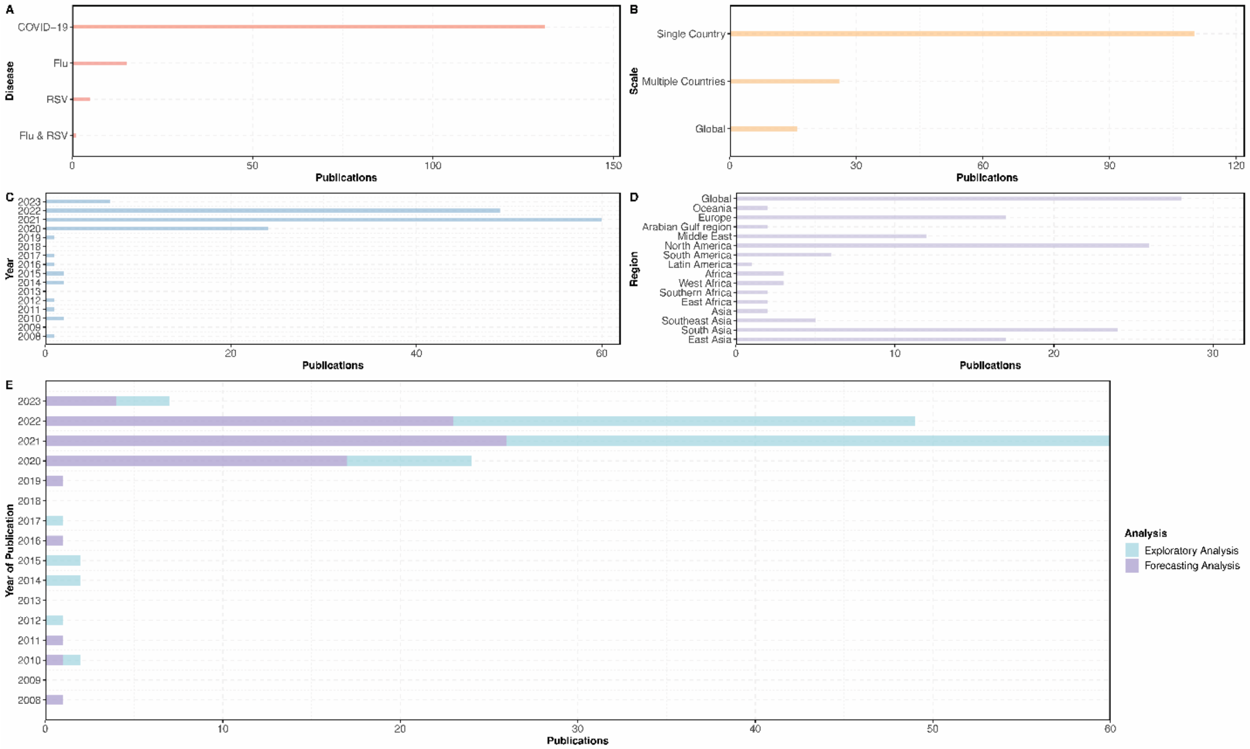

Figure S1. General characteristics of the identified scenario literature. **Panel A.** Publications grouped by disease. **Panel B.** Publications grouped by country-scale. **Panel C.** Publications grouped by published year. **Panel D.** Publications grouped by study region. **Panel E.** Publications grouped by both published year and analysis type.

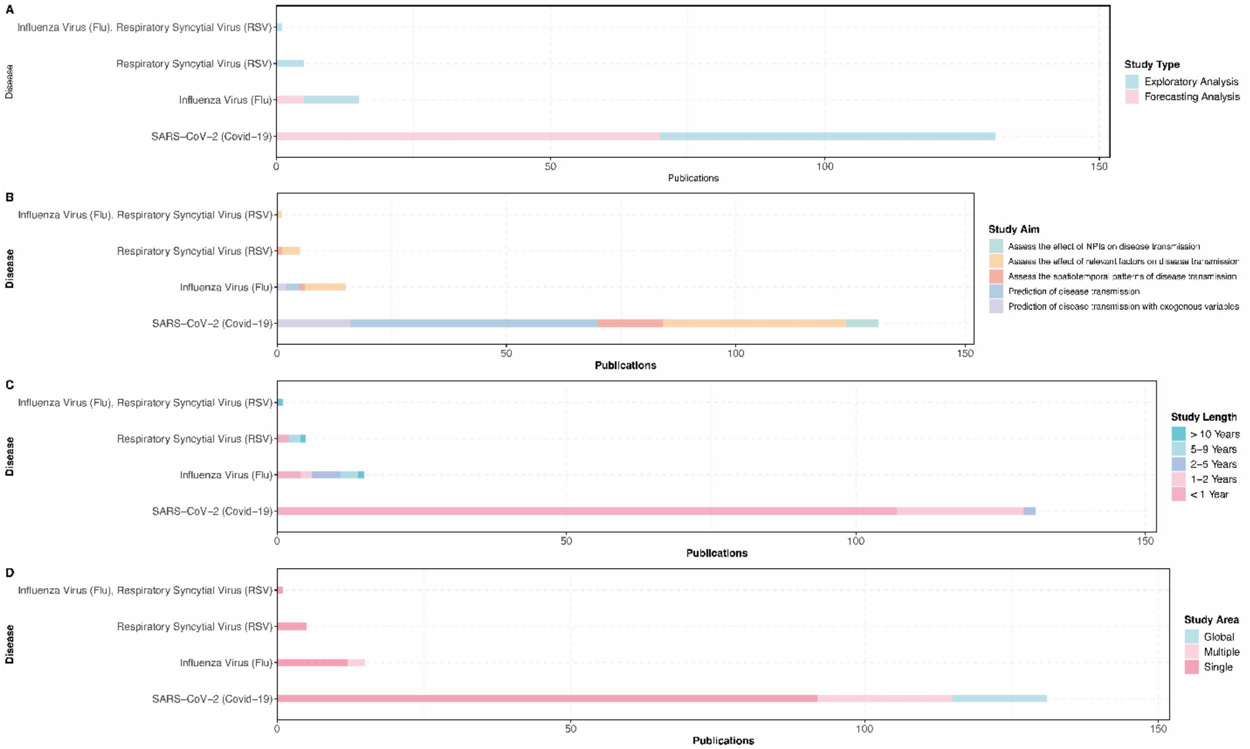

Figure S2. General characteristics of the identified scenario literature by disease. **Panel A.** Publications grouped by study type. **Panel B.** Publications grouped by study aim. **Panel C.** Publications grouped by length of study. **Panel D.** Publications grouped by study area.

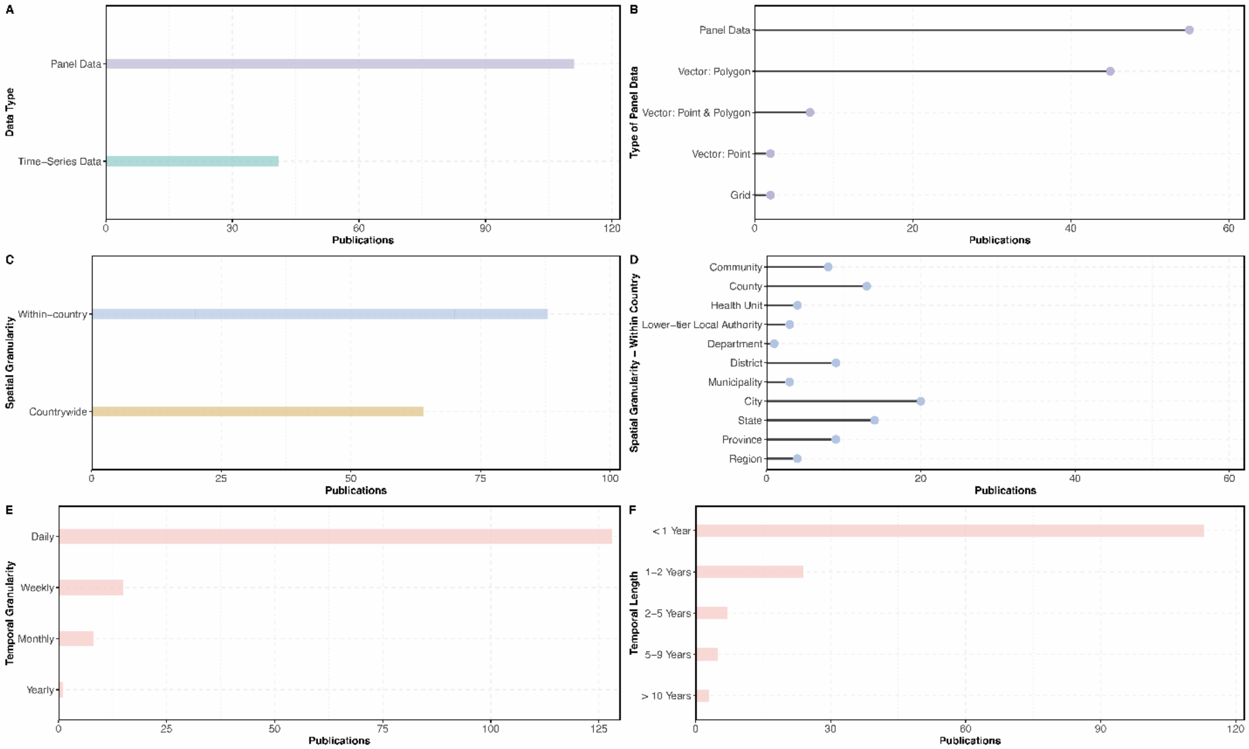

Figure 3. Characteristics of data in the identified scenario literature. **Panel A.** Publications grouped by data type. **Panel B.** Publications grouped by data type in panel data. **Panel C.** Publications grouped by spatial granularity of data. **Panel D.** Publications grouped by spatial granularity of data within country scale. **Panel E.** Publications grouped by length of study data. **Panel F.** Publications grouped by temporal granularity of study data.

Reference

[1] Niraula P, Mateu J, Chaudhuri S. A Bayesian machine learning approach for spatio-temporal prediction of COVID-19 cases. Stochastic Environmental Research and Risk Assessment. 2022;36(8):2265–2283.

[2] Borghi PH, Zakordonets O, Teixeira JP, et al. A COVID-19 time series forecasting model based on MLP ANN. International Conference on ENTERprise Information Systems (CENTERIS) / International Conference on Project MANagement (ProjMAN) / International Conference on Health and Social Care Information Systems and Technologies (HCist). 2020;181:940–947.

[3] Rahman Md S, Chowdhury AH. A data-driven eXtreme gradient boosting machine learning model to predict COVID-19 transmission with meteorological drivers. PLoS ONE [Internet]. 2022;17(9 September) (no pagination).

[4] Zheng HL, An SY, Qiao BJ, et al. A data-driven interpretable ensemble framework based on tree models for forecasting the occurrence of COVID-19 in the USA. Environmental science and pollution research international. 2023;30(5):13648–13659.

[5] Shahin AI, Almotairi S. A Deep Learning BiLSTM Encoding-Decoding Model for COVID-19 Pandemic Spread Forecasting. Fractal and Fractional. 2021;5(4):27.

[6] Alzahrani SM. A log linear Poisson autoregressive model to understand COVID-19 dynamics in Saudi Arabia. Beni-Suef University Journal of Basic and Applied Sciences. 2022;11(1):6.

[7] Sahai SY, Gurukar S, KhudaBukhsh WR, et al. A machine learning model for nowcasting epidemic incidence. Mathematical Biosciences [Internet]. 2022;343 (no pagination).

[8] Lucas B, Vahedi B, Karimzadeh M. A spatiotemporal machine learning approach to forecasting COVID-19 incidence at the county level in the USA. International Journal of Data Science and Analytics. :20.

[9] Adiga A, Wang LJ, Hurt B, et al. All Models Are Useful: Bayesian Ensembling for Robust High Resolution COVID-19 Forecasting. 27th ACM SIGKDD International Conference on Knowledge Discovery and Data Mining (KDD). 2021;2505–2513.

[10] Lakshmanarao A, Babu MR, Kiran TSR. An Efficient Covid19 Epidemic Analysis and Prediction Model Using Machine Learning Algorithms. International Journal of Online and Biomedical Engineering. 2021;17(11):176–184.

[11] El-Din Saad NG, Ghoniemy S, Faheem H, et al. An Evaluation of Time Series-Based Modeling and Forecasting of Infectious Diseases Progression using Statistical Versus Compartmental Methods. 5th International Conference on Computing and Informatics (IEEE ICCI). 2022;263–273.

[12] Ahmad HF, Khaloofi H, Azhar Z, et al. An Improved COVID-19 Forecasting by Infectious Disease Modelling Using Machine Learning. Applied Sciences-Basel. 2021;11(23):38.

[13] Shoko C, Njuho P. Arima Model in Predicting of Covid-19 Epidemic for the Southern Africa Region. African Journal of Infectious Diseases. 2023;17(1):1–9.

[14] Mollalo A, Rivera KM, Vahedi B. Artificial neural network modeling of novel coronavirus (COVID-19) incidence rates across the continental United States. International Journal of Environmental Research and Public Health. 2020;17(12):1–13.

[15] Dash S, Chakraborty C, Giri SK, et al. BIFM: Big-Data Driven Intelligent Forecasting Model for COVID-19. Ieee Access. 2021;9:97505–97517.

[16] Fritz C, Dorigatti E, Rugamer D. Combining graph neural networks and spatio-temporal disease models to improve the prediction of weekly COVID-19 cases in Germany. Scientific reports. 2022;12(1):3930.

[17] Qureshi M, Daniyal M, Tawiah K. Comparative Evaluation of the Multilayer Perceptron Approach with Conventional ARIMA in Modeling and Prediction of COVID-19 Daily Death Cases. Journal of Healthcare Engineering. 2022;2022:4864920.

[18] Dairi A, Harrou F, Zeroual A, et al. Comparative study of machine learning methods for COVID-19 transmission forecasting. Journal of Biomedical Informatics [Internet]. 2021;118 (no pagination).

[19] Makinde OS, Adeola AM, Abiodun GJ, et al. Comparison of Predictive Models and Impact Assessment of Lockdown for COVID-19 over the United States. Journal of epidemiology and global health [Internet]. 2021;22.

[20] Basu S, Sen S. COVID 19 Pandemic, Socio-Economic Behaviour and Infection Characteristics: An Inter-Country Predictive Study Using Deep Learning. Computational Economics. :32.

[21] Contreras-Espinoza S, Novoa-Munoz F, Blazsek S, et al. COVID-19 Active Case Forecasts in Latin American Countries Using Score-Driven Models. Mathematics. 2023;11(1):17.

[22] da Silva CC, de Lima CL, da Silva ACG, et al. Covid-19 Dynamic Monitoring and Real-Time Spatio-Temporal Forecasting. Frontiers in public health. 2021;9:641253.

[23] Masum AM, Khushbu SA, Keya M, et al. COVID-19 in Bangladesh: A Deeper Outlook into The Forecast with Prediction of Upcoming Per Day Cases Using Time Series. 9th International Young Scientists Conference in Computational Science (YSC). 2020;178:291–300.

[24] Yahya BM, Yahya FS, Thannoun RG. COVID-19 prediction analysis using artificial intelligence procedures and GIS spatial analyst: a case study for Iraq. Applied Geomatics. 2021;13(3):481–491.

[25] Gomez-Cravioto DA, Diaz-Ramos RE, Cantu-Ortiz FJ, et al. Data Analysis and Forecasting of the COVID-19 Spread: A Comparison of Recurrent Neural Networks and Time Series Models. Cognitive Computation. :12.

[26] Ramirez-Alcocer UM, Tello-Leal E, Macias-Hernandez BA, et al. Data-Driven Prediction of COVID-19 Daily New Cases through a Hybrid Approach of Machine Learning Unsupervised and Deep Learning. Atmosphere. 2022;13(8):20.

[27] Chandra R, Jain A, Chauhan DS. Deep learning via LSTM models for COVID-19 infection forecasting in India. PLoS ONE [Internet]. 2022;17(1 January) (no pagination).

[28] Munoz-Organero M, Queipo-Alvarez P. Deep Spatiotemporal Model for COVID-19 Forecasting. Sensors [Internet]. 2022;22(9).

[29] Pan Y, Zhang LM, Yan ZZ, et al. Discovering optimal strategies for mitigating COVID-19 spread using machine learning: Experience from Asia. Sustainable Cities and Society. 2021;75:16.

[30] Canino MP, Cesario E, Vinci A, et al. Epidemic forecasting based on mobility patterns: an approach and experimental evaluation on COVID-19 Data. Social Network Analysis and Mining. 2022;12(1):15.

[31] Awwad FA, Mohamoud MA, Abonazel MR. Estimating COVID-19 cases in Makkah region of Saudi Arabia: Space-time ARIMA modeling. PLoS ONE [Internet]. 2021;16(4 April) (no pagination).

[32] Wang YB, Xu CJ, Yao SQ, et al. Estimating the COVID-19 prevalence and mortality using a novel data-driven hybrid model based on ensemble empirical mode decomposition. Scientific Reports. 2021;11(1):17.

[33] Ceylan Z. Estimation of COVID-19 prevalence in Italy, Spain, and France. Science of the Total Environment. 2020;729:7.

[34] Sabry I, Mourad AHI, Idrisi AH, et al. Forecasting COVID-19 Cases in Egypt Using ARIMA-Based Time-Series Analysis. Eurasian Journal of Medicine and Oncology. 2021;5(2):123–131.

[35] Nguyen HM, Turk PJ, McWilliams AD. Forecasting COVID-19 Hospital Census: A Multivariate Time-Series Model Based on Local Infection Incidence. Jmir Public Health and Surveillance. 2021;7(8):13.

[36] Xu L, Magar R, Barati Farimani A. Forecasting COVID-19 new cases using deep learning methods. Computers in Biology and Medicine [Internet]. 2022;144 (no pagination).

[37] Khedhiri S. Forecasting COVID-19 infections in the Arabian Gulf region. Modeling Earth Systems and Environment. 2022;8(3):3813–3822.

[38] Khan F, Ali S, Saeed A, et al. Forecasting daily new infections, deaths and recovery cases due to COVID-19 in Pakistan by using Bayesian Dynamic Linear Models. PLoS ONE [Internet]. 2021;16(6 June) (no pagination).

[39] Sulasikin A, Nugraha Y, Kanggrawan J, et al. Forecasting for a data-driven policy using time series methods in handling COVID-19 pandemic in Jakarta. IEEE International Smart Cities Conference (ISC2) [Internet]. 2020;

[40] Wang Y, Xu C, Yao S, et al. Forecasting the epidemiological trends of COVID-19 prevalence and mortality using the advanced alpha-Sutte Indicator. Epidemiology & Infection. 2020;148:e236.

[41] Kibria HB, Jyoti O, Matin A. Forecasting the spread of the third wave of COVID-19 pandemic using time series analysis in Bangladesh. Informatics in Medicine Unlocked [Internet]. 2022;28 (no pagination).

[42] Ilie OD, Cojocariu RO, Ciobica A, et al. Forecasting the Spreading of COVID-19 across Nine Countries from Europe, Asia, and the American Continents Using the ARIMA Models. Microorganisms. 2020;8(8):18.

[43] Chew AWZ, Pan Y, Wang Y, et al. Hybrid deep learning of social media big data for predicting the evolution of COVID-19 transmission. Knowledge-Based Systems. 2021;233:21.

[44] Chumachenko D, Meniailov I, Bazilevych K, et al. Investigation of Statistical Machine Learning Models for COVID-19 Epidemic Process Simulation: Random Forest, K-Nearest Neighbors, Gradient Boosting. Computation. 2022;10(6):22.

[45] Acosta MFJ, Garcia-Zapirain B, Ieee, et al. Machine Learning Algorithms for Forecasting COVID 19 Confirmed Cases in America. IEEE International Symposium on Signal Processing and Information Technology (ISSPIT) [Internet]. 2020; doi: 10.1109/isspit51521.2020.9408742.

[46] Soebiyanto RP, Adimi F, Kiang RK. Modeling and Predicting Seasonal Influenza Transmission in Warm Regions Using Climatological Parameters. Plos One. 2010;5(3):10.

[47] Ankarali H, Erarslan N, Pasin O, et al. Modeling and Short-Term Forecasts of Indicators for COVID-19 Outbreak in 25 Countries at the end of March. Bangladesh Journal of Medical Science. 2020;19:S6–S20.
[48] Gning L, Ndour C, Tchuenche JM. Modeling COVID-19 daily cases in Senegal using a generalized Waring regression model. Physica a-Statistical Mechanics and Its Applications. 2022;597:10.
[49] Giuliani D, Dickson MM, Espa G, et al. Modelling and predicting the spatio-temporal spread of COVID-19 in Italy. BMC Infectious Diseases [Internet]. 2020;20(1) (no pagination).
[50] Benth JS, Hofoss D. Modelling and prediction of weekly incidence of influenza A specimens in England and Wales. Epidemiology and Infection. 2008;136(12):1658–1666.
[51] Ibrahim Z, Tulay P, Abdullahi J. Multi-region machine learning-based novel ensemble approaches for predicting COVID-19 pandemic in Africa. Environmental science and pollution research international. 2023;30(2):3621–3643.
[52] Meintrup D, Nowak-Machen M, Borgmann S. Nine months of COVID-19 pandemic in europe: A comparative time series analysis of cases and fatalities in 35 countries. International Journal of Environmental Research and Public Health [Internet]. 2021;18(12) (no pagination).
[53] Arun SS, Iyer GN, Ieee, et al. On the Analysis of COVID19-Novel Corona Viral Disease Pandemic Spread Data Using Machine Learning Techniques. International Conference on Intelligent Computing and Control Systems (ICICCS). 2020;1222–1227.
[54] Khuhawar UZ, Siddiqui IF, Arain QA, et al. On-Ground Distributed COVID-19 Variant Intelligent Data Analytics for a Regional Territory. Wireless Communications & Mobile Computing. 2021;2021:19.
[55] Ogunjo ST, Fuwape IA, Rabiu AB. Predicting COVID-19 Cases From Atmospheric Parameter Using Machine Learning Approach. Geohealth. 2022;6(4):8.
[56] Zhang Y, Yakob L, Bonsall MB, et al. Predicting seasonal influenza epidemics using cross-hemisphere influenza surveillance data and local internet query data. Scientific reports. 2019;9(1):3262.
[57] Barria-Sandoval C, Ferreira G, Benz-Parra K, et al. Prediction of confirmed cases of and deaths caused by COVID-19 in Chile through time series techniques: A comparative study. Plos One. 2021;16(4):16.
[58] Gupta AK, Singh V, Mathur P, et al. Prediction of COVID-19 pandemic measuring criteria using support vector machine, prophet and linear regression models in Indian scenario. Journal of Interdisciplinary Mathematics. 2021;24(1):89–108.
[59] Ilu SY, Rajesh P, Mohammed H. Prediction of COVID-19 using long short-term memory by integrating principal component analysis and clustering techniques. Informatics in Medicine Unlocked [Internet]. 2022;31 (no pagination).
[60] Amar LA, Taha AA, Mohamed MY. Prediction of the final size for COVID-19 epidemic using machine learning: A case study of Egypt. Infectious Disease Modelling. 2020;5:622–634.
[61] Daniyal M, Ogundokun RO, Abid K, et al. Predictive modeling of COVID-19 death cases in Pakistan. Infectious Disease Modelling. 2020;5:897–904.
[62] Peng Y, Li C, Rong Y, et al. Real-time prediction of the daily incidence of COVID-19 in 215 countries and territories using machine learning: Model development and validation. Journal of Medical Internet Research [Internet]. 2021;23(6) (no pagination).
[63] Gera S, Mridul M, Joshi MK, et al. Regression Analysis And Future Forecasting Of COVID-19 Using Machine Learnings Algorithm. 11th International Conference on Cloud Computing, Data Science and Engineering (Confluence). 2021;1014–1018.
[64] Satu MS, Howlader KC, Mahmud M, et al. Short-Term Prediction of COVID-19 Cases Using Machine Learning Models. Applied Sciences-Basel. 2021;11(9):18.
[65] Naeem M, Mashwani WK, Abiad M, et al. Soft computing techniques for forecasting of COVID-19 in Pakistan. Alexandria Engineering Journal. 2023;63:45–56.
[66] Roy S, Bhunia GS, Shit PK. Spatial prediction of COVID-19 epidemic using ARIMA techniques in India. Modeling Earth Systems and Environment. 2021;7(2):1385–1391.
[67] Nikparvar B, Rahman MM, Hatami F, et al. Spatio-temporal prediction of the COVID-19 pandemic in US counties: modeling with a deep LSTM neural network. Scientific reports. 2021;11(1):21715.
[68] Takele R. Stochastic modelling for predicting COVID-19 prevalence in East Africa Countries. Infectious Disease Modelling. 2020;5:598–607.
[69] Ismail L, Materwala H, Znati T, et al. Tailoring time series models for forecasting coronavirus spread: Case studies of 187 countries. Computational and Structural Biotechnology Journal. 2020;18:2972–3206.
[70] Ilie OD, Ciobica A, Doroftei B. Testing the Accuracy of the ARIMA Models in Forecasting the Spreading of COVID-19 and the Associated Mortality Rate. Medicina [Internet]. 2020;56(11).

[71] Babu MA, Ahmmed MM, Abu Helal M, et al. The FBProphet forecasting model to evaluate the spread of COVID-19 pandemic: A machine learning approach. Journal of Interdisciplinary Mathematics. 2022;25(7):2073–2082.
[72] Semwal J, Bahuguna A, Sharma N, et al. Time Series Analysis of COVID-19 Data- A study from Northern India. Indian Journal of Community Health. 2022;34(2):202–206.
[73] Song X, Xiao J, Deng J, et al. Time series analysis of influenza incidence in Chinese provinces from 2004 to 2011. Medicine (United States) [Internet]. 2016;95(26) (no pagination).
[74] Zrieq R, Kamel S, Boubaker S, et al. Time-Series Analysis and Healthcare Implications of COVID-19 Pandemic in Saudi Arabia. Healthcare. 2022;10(10):27.
[75] Spaeder MC, Stroud JR, Song X. Time-series model to predict impact of H1N1 influenza on a children's hospital. Epidemiology & Infection. 2012;140(5):798–802.
[76] Neelon B, Wen CC, Benjamin-Neelon SE. A multivariate spatiotemporal model for tracking COVID-19 incidence and death rates in socially vulnerable populations. Journal of Applied Statistics. :24.
[77] Minu RI, Nagarajan G. A Statistical Non-Parametric data analysis for COVID-19 incidence data. ISA transactions. 2022;130:675–683.
[78] Jaya I, Folmer H. Bayesian spatiotemporal forecasting and mapping of COVID-19 risk with application to West Java Province, Indonesia. Journal of Regional Science. 2021;61(4):849–881.
[79] Zheng Z, Pitzer VE, Warren JL, et al. Community factors associated with local epidemic timing of respiratory syncytial virus: A spatiotemporal modeling study. Science advances [Internet]. 2021;7(26).
[80] Antonio N, Rita P, Saraiva P. COVID-19: Worldwide Profiles during the First 250 Days. Applied Sciences-Basel. 2021;11(8):21.
[81] Guerra OD, Salcines VC, Prieto DC. Data mining and socio-spatial patterns of COVID-19: geo-prevention keys for tackling the pandemic. Boletin De La Asociacion De Geografos Espanoles. 2021;(91):1–40.
[82] Niu B, Liang R, Zhang S, et al. Epidemic analysis of COVID-19 in Italy based on spatiotemporal geographic information and Google Trends. Transboundary and Emerging Diseases. 2021;68(4):2384–2400.
[83] Tang C, Wang TD, Zhang PP. Functional data analysis: An application to COVID-19 data in the United States in 2020. Quantitative Biology. 2022;10(2):172–187.
[84] Husnayain A, Chuang TW, Fuad A, et al. High variability in model performance of Google relative search volumes in spatially clustered COVID-19 areas of the USA. International Journal of Infectious Diseases. 2021;109:269–278.
[85] Asem N, Ramadan A, Hassany M, et al. Pattern and determinants of COVID-19 infection and mortality across countries: An ecological study. Heliyon. 2021;7(7):8.
[86] Huang D, Dong W, Wang Q. Spatial and temporal analysis of human infection with the avian influenza A (H7N9) virus in China and research on risk assessment agent-based model. International journal of infectious diseases : IJID : official publication of the International Society for Infectious Diseases [Internet]. 2021;12.
[87] Tabasi M, Alesheikh AA, Kalantari M, et al. Spatial Modeling of COVID-19 Prevalence Using Adaptive Neuro-Fuzzy Inference System. Isprs International Journal of Geo-Information. 2022;11(10):14.
[88] Ngwira A, Kumwenda F, Munthali ECS, et al. Spatial temporal distribution of COVID-19 risk during the early phase of the pandemic in Malawi. Peerj. 2021;9:15.
[89] Al-Kindi KM, Alkharusi A, Alshukaili D, et al. Spatiotemporal Assessment of COVID-19 Spread over Oman Using GIS Techniques. Earth Systems and Environment. 2020;4(4):797–811.
[90] Nightingale ES, Abbott S, Russell TW, et al. The local burden of disease during the first wave of the COVID-19 epidemic in England: estimation using different data sources from changing surveillance practices. Bmc Public Health. 2022;22(1):14.
[91] Bag R, Ghosh M, Biswas B, et al. Understanding the spatio-temporal pattern of COVID-19 outbreak in India using GIS and India's response in managing the pandemic. Regional Science Policy and Practice. 2020;12(6):1063–1103.
[92] Al-Sharari W, Mahmood MA, Abd El-Aziz AA, et al. A Detecting Technique for the Climatic Factors that Aided the Spread of COVID-19 using Deep and Machine Learning Algorithms. International Journal of Computer Science and Network Security. 2022;22(6):131–138.
[93] Shrivastav LK, Jha SK. A gradient boosting machine learning approach in modeling the impact of temperature and humidity on the transmission rate of COVID-19 in India. Applied Intelligence. 2021;51(5):2727–2739.
[94] de Souza APG, de Miranda Mota CM, Rosa AGF, et al. A spatial-temporal analysis at the early stages of the COVID-19 pandemic and its determinants: The case of Recife neighborhoods, Brazil. PLoS ONE [Internet]. 2022;17(5 May) (no pagination).

[95] Paez A, Lopez FA, Menezes T, et al. A Spatio-Temporal Analysis of the Environmental Correlates of COVID-19 Incidence in Spain. Geographical Analysis. 2021;53(3):397–421.
[96] Thayer WM, Hasan MZ, Sankhla P, et al. An interrupted time series analysis of the lockdown policies in India: a national-level analysis of COVID-19 incidence. Health policy and planning. 2021;36(5):620–629.
[97] Ardakani SP, Xia TQ, Cheshmehzangi A, et al. An urban-level prediction of lockdown measures impact on the prevalence of the COVID-19 pandemic. Genus. 2022;78(1):17.
[98] Wu C, Zhou MJ, Liu PY, et al. Analyzing COVID-19 Using Multisource Data: An Integrated Approach of Visualization, Spatial Regression, and Machine Learning. Geohealth. 2021;5(8):14.
[99] Barria-Sandoval C, Ferreira G, Lagos B, et al. Assessing the effectiveness of quarantine measures during the COVID-19 pandemic in Chile using Bayesian structural time series models. Infectious Disease Modelling. 2022;7(4):625–636.
[100] Chattopadhyay A, Shaw S. Association Between Air Pollution and COVID-19 Pandemic: An Investigation in Mumbai, India. Geohealth. 2021;5(7):16.
[101] Gujral H, Sinha A. Association between exposure to airborne pollutants and COVID-19 in Los Angeles, United States with ensemble-based dynamic emission model. Environmental Research [Internet]. 2021;194 (no pagination).
[102] Yuan J, Wu Y, Jing W, et al. Association between meteorological factors and daily new cases of COVID-19 in 188 countries: A time series analysis. Science of the Total Environment [Internet]. 2021;780 (no pagination).
[103] Weinberger DM, Klugman KP, Steiner CA, et al. Association between Respiratory Syncytial Virus Activity and Pneumococcal Disease in Infants: A Time Series Analysis of US Hospitalization Data. PLoS Medicine. 2015;12(1):1–12.
[104] Shimmei K, Nakamura T, Ng CFS, et al. Association Between Seasonal Influenza and Absolute Humidity: Time-Series Analysis with Daily Surveillance Data in Japan. Scientific reports. 2020;10(1):7764.
[105] Chen Z, Zhu Y, Wang Y, et al. Association of meteorological factors with childhood viral acute respiratory infections in subtropical China: An analysis over 11 years. Archives of Virology. 2014;159(4):631–639.
[106] Dlamini WMD, Simelane SP, Nhlabatsi NM. Bayesian network-based spatial predictive modelling reveals COVID-19 transmission dynamics in Eswatini. Spatial Information Research. 2022;30(1):183–194.
[107] dos Santos JMA, de Menezes TA, de Arruda RG, et al. Climate influences on COVID-19 prevalence rates: An application of a panel data spatial model. Regional Science Policy and Practice. :18.
[108] Zhang R, Lai KY, Liu W, et al. Community-level ambient fine particulate matter and seasonal influenza among children in Guangzhou, China: A Bayesian spatiotemporal analysis. Science of the Total Environment [Internet]. 2022;826 (no pagination).
[109] Tang JW, Lai FY, Nymadawa P, et al. Comparison of the incidence of influenza in relation to climate factors during 2000-2007 in five countries. Journal of medical virology. 2010;82(11):1958–1965.
[110] Batool H, Tian LX. Correlation Determination between COVID-19 and Weather Parameters Using Time Series Forecasting: A Case Study in Pakistan. Mathematical Problems in Engineering. 2021;2021:9.
[111] Qi H, Xiao S, Shi R, et al. COVID-19 transmission in Mainland China is associated with temperature and humidity: A time-series analysis. Science of the Total Environment [Internet]. 2020;728 (no pagination).
[112] Wagatsuma K, Koolhof IS, Shobugawa Y, et al. Decreased human respiratory syncytial virus activity during the COVID-19 pandemic in Japan: an ecological time-series analysis. BMC Infectious Diseases [Internet]. 2021;21(1) (no pagination).
[113] He S, Lee J, Langworthy B, et al. Delay in the Effect of Restricting Community Mobility on the Spread of COVID-19 during the First Wave in the United States. Open Forum Infectious Diseases [Internet]. 2022;9(1) (no pagination).
[114] Hu W, Williams G, Phung H, et al. Did socio-ecological factors drive the spatiotemporal patterns of pandemic influenza A (H1N1)? Environment International. 2012;45:39–43.
[115] Alimohamadi Y, Holakouie-Naieni K, Sepandi M, et al. Effect of social distancing on COVID-19 incidence and mortality in Iran since february 20 to May 13, 2020: An interrupted time series analysis. Risk Management and Healthcare Policy. 2020;13:1695–1700.
[116] Huang X, Mengersen K, Milinovich G, et al. Effect of weather variability on seasonal influenza among different age groups in Queensland, Australia: A Bayesian spatiotemporal analysis. Journal of Infectious Diseases. 2017;215(11):1695–1701.
[117] James A, Malagon-Morris R, Gurusinghe S, et al. Evaluating the impact of social distancing on COVID-19 hospitalizations using interrupted time series regression. International Journal of Academic Medicine. 2022;8(1):24–31.

[118] Chakraborti S, Maiti A, Pramanik S, et al. Evaluating the plausible application of advanced machine learnings in exploring determinant factors of present pandemic: A case for continent specific COVID-19 analysis. Science of the Total Environment [Internet]. 2021;765 (no pagination).
[119] Ma SP, Zhang XL, Wang K, et al. Exploring the risk factors of COVID-19 Delta variant in the United States based on Bayesian spatio-temporal analysis. Transboundary and Emerging Diseases. 2022;69(5):E2731–E2744.
[120] Gaudart J, Landier J, Huiart L, et al. Factors associated with the spatial heterogeneity of the first wave of COVID-19 in France: a nationwide geo-epidemiological study. Lancet Public Health. 2021;6(4):E222–E231.
[121] Hassaan MA, Abdelwahab RG, Elbarky TA, et al. GIS-Based Analysis Framework to Identify the Determinants of COVID-19 Incidence and Fatality in Africa. Journal of primary care & community health. 2021;12:21501327211041208.
[122] Ben S, Xin J, Chen S, et al. Global internet search trends related to gastrointestinal symptoms predict regional COVID-19 outbreaks. Journal of Infection. 2022;84(1):56–63.
[123] Dayaratna KD, Gonshorowski D, Kolesar M. Hierarchical Bayesian spatio-temporal modeling of COVID-19 in the United States. Journal of Applied Statistics. :18.
[124] Nassiri H, Mohammadpour SI, Dahaghin M. How do the smart travel ban policy and intercity travel pattern affect COVID-19 trends? Lessons learned from Iran. PLoS ONE [Internet]. 2022;17(10 October) (no pagination).
[125] Nazia N, Law J, Butt ZA. Identifying spatiotemporal patterns of COVID-19 transmissions and the drivers of the patterns in Toronto: a Bayesian hierarchical spatiotemporal modelling. Scientific reports. 2022;12(1):9369.
[126] Panigrahi A, Mohapatra I, Kanyari SS, et al. Impact of environmental temperature and relative humidity on spread of COVID-19 infection in India: a cross-sectional time-series analysis. Archives of Environmental and Occupational Health. 2022;77(5):389–395.
[127] Ademu LO, Gao J, Thompson OP, et al. Impact of Short-Term Air Pollution on Respiratory Infections: A Time-Series Analysis of COVID-19 Cases in California during the 2020 Wildfire Season. International Journal of Environmental Research and Public Health [Internet]. 2022;19(9) (no pagination).
[128] Gurram MK, Wang MX, Wang YC, et al. Impact of urbanisation and environmental factors on spatial distribution of COVID-19 cases during the early phase of epidemic in Singapore. Scientific Reports. 2022;12(1):15.
[129] Ganegoda NC, Wijaya KP, Amadi M, et al. Interrelationship between daily COVID-19 cases and average temperature as well as relative humidity in Germany. Scientific reports. 2021;11(1):11302.
[130] Chien LC, Chen LWA, Lin RT. Lagged meteorological impacts on COVID-19 incidence among high-risk counties in the United States-a spatiotemporal analysis. Journal of exposure science & environmental epidemiology. 2022;32(5):774–781.
[131] Zhang H, Wen S, Zheng J, et al. Meteorological factors affecting respiratory syncytial virus infection: A time-series analysis. Pediatric pulmonology [Internet]. 2020;07.
[132] Yin C, Zhao W, Pereira P. Meteorological factors' effects on COVID-19 show seasonality and spatiality in Brazil. Environmental Research [Internet]. 2022;208 (no pagination).
[133] Madden JM, More S, Teljeur C, et al. Population Mobility Trends, Deprivation Index and the Spatio-Temporal Spread of Coronavirus Disease 2019 in Ireland. International Journal of Environmental Research and Public Health. 2021;18(12):16.
[134] Hassan MS, Bhuiyan MAH, Tareq F, et al. Relationship between COVID-19 infection rates and air pollution, geo-meteorological, and social parameters. Environmental Monitoring and Assessment [Internet]. 2021;193(1) (no pagination).
[135] Dong KQ, Guo L. Research on the Spatial Correlation and Spatial Lag of COVID-19 Infection Based on Spatial Analysis. Sustainability. 2021;13(21):16.
[136] Thongpan I, Vongpunsawad S, Poovorawan Y. Respiratory syncytial virus infection trend is associated with meteorological factors. Scientific reports. 2020;10(1):10931.
[137] Chen FF, Liu Z, Huang T, et al. Short-Term Effects of Air Pollution on the Risk of Influenza in Jinan, China during 2020-2021: A Time-Series Analysis. Atmosphere. 2023;14(1):11.
[138] Meng Y, Lu Y, Xiang H, et al. Short-term effects of ambient air pollution on the incidence of influenza in Wuhan, China: A time-series analysis. Environmental Research [Internet]. 2021;192 (no pagination).
[139] Huang XC, Zhou H, Yang XF, et al. Spatial Characteristics of Coronavirus Disease 2019 and Their Possible Relationship With Environmental and Meteorological Factors in Hubei Province, China. Geohealth. 2021;5(6):9.

[140] Barbosa B, Silva M, Capinha C, et al. Spatial correlates of COVID-19 first wave across continental Portugal. Geospatial Health [Internet]. 2022;17(s1).
[141] Fatholahi SN, Pan C, Wang LY, et al. SPATIAL MODELLING OF COVID-19 INCIDENCE RATE IN CANADA. 24th ISPRS Congress on Imaging Today, Foreseeing Tomorrow. 2022;43-B4:111–116.
[142] Jana A, Kundu S, Shaw S, et al. Spatial shifting of COVID-19 clusters and disease association with environmental parameters in India: A time series analysis. Environmental Research [Internet]. 2023;222 (no pagination).
[143] Kianfar N, Mesgari MS, Mollalo A, et al. Spatio-temporal modeling of COVID-19 prevalence and mortality using artificial neural network algorithms. Spatial and Spatio-Temporal Epidemiology. 2022;40:16.
[144] Aral N, Bakir H. Spatiotemporal Analysis of Covid-19 in Turkey. Sustainable Cities and Society. 2022;76:10.
[145] Nottmeyer L, Armstrong B, Lowe R, et al. The association of COVID-19 incidence with temperature, humidity, and UV radiation - A global multi-city analysis. Science of the Total Environment [Internet]. 2023;854 (no pagination).
[146] Hass FS, Arsanjani JJ. The geography of the covid-19 pandemic: A data-driven approach to exploring geographical driving forces. International Journal of Environmental Research and Public Health. 2021;18(6):1–19.
[147] Molefi M, Tlhakanelo JT, Phologolo T, et al. The Impact of China’s Lockdown Policy on the Incidence of COVID-19: An Interrupted Time Series Analysis. BioMed Research International [Internet]. 2021;2021 (no pagination).
[148] He Z, Chin Y, Yu S, et al. The Influence of Average Temperature and Relative Humidity on New Cases of COVID-19: Time-Series Analysis. JMIR public health and surveillance. 2021;7(1):e20495.
[149] Liu M, Li Z, Zhu Y, et al. The spatial clustering analysis of COVID-19 and its associated factors in mainland China at the prefecture level. Science of the Total Environment [Internet]. 2021;777 (no pagination).
[150] Lin QW, Ou GL, Wang RY, et al. The Spatiotemporal Characteristics and Climatic Factors of COVID-19 in Wuhan, China. Sustainability. 2021;13(14):17.
[151] Imai C, Brooks WA, Chung Y, et al. Tropical influenza and weather variability among children in an urban low-income population in Bangladesh. Global health action. 2014;7:24413.
[152] Hu W, Zhang W, Huang X, et al. Weather variability and influenza A (H7N9) transmission in Shanghai, China: A Bayesian spatial analysis. Environmental Research. 2015;136:405–412.
